# Malnutrition diagnosed by the Global Leadership Initiative on Malnutrition criteria predicting survival and clinical outcomes of patients with cancer: A systematic review and meta-analysis

**DOI:** 10.3389/fnut.2022.1053165

**Published:** 2022-12-06

**Authors:** Dadi Peng, Kezhen Zong, Hang Yang, Zuotian Huang, Tong Mou, Puen Jiang, Zhongjun Wu

**Affiliations:** ^1^Department of Hepatobiliary Surgery, The First Affiliated Hospital of Chongqing Medical University, Chongqing, China; ^2^Department of Hepatobiliary Pancreatic Tumor Center, Chongqing University Cancer Hospital, Chongqing, China

**Keywords:** Global Leadership Initiative on Malnutrition, malnutrition, cancer, survival, clinical outcomes, complications, readmission

## Abstract

**Objectives:**

Recently, some cohorts have looked into the use of Global Leadership Initiative on Malnutrition (GLIM) criteria in cancer patients. The objective of the current meta-analysis was to determine its utility in predicting clinical and survival outcomes for cancer patients.

**Method:**

Searching and screening literature from PubMed, Web of Science and Embase until September 13, 2022 was performed by two researchers independently. According to the exclusion and inclusion criteria, articles reporting the impact of malnutrition diagnosed by GLIM on long-term survival and clinical outcomes were included. Data of interest were also extracted from the included papers. The stability of the pooled results was evaluated using sensitivity analysis. With the aid of subgroup analysis, heterogeneity was revealed. To assess publication bias, Egger’s and Begg’s tests were conducted. The influence of publication bias on the pooling risk estimate was examined using a trim-and-fill analysis.

**Results:**

15 studies that qualified for our study were identified. Pooled hazard ratio (HR) from both multivariate and univariate regression analysis showed a worse overall survival in GLIM-defined malnourished cancer patients than those in well-nourished status. Meanwhile, disease-free survival was also poorer in malnourished patients. Moreover, pooled odds ratio (OR) demonstrated that malnourished cancer patients were more likely to develop overall postoperative complications, complications ≥ Clavien-Dindo grade IIa and complications ≥ Clavien-Dindo grade IIIa. Two articles reported negative relation between GLIM-defined malnutrition and 30-day readmission/mortality.

**Conclusion:**

GLIM-defined malnutrition possesses value in predicting poorer survival and clinical outcomes for cancer patients.

**Systematic review registration:**

[https://www.crd.york.ac.uk/PROSPERO/display_record.php?RecordID=321094], identifier [CRD42022321094].

## Introduction

As the nature of cancer disorients the immunological and metabolic condition, studies have revealed that malnutrition is prevalent in cancer patients, with its occurrence fluctuating owing to factors including patients’ characteristics, tumor types and criteria applied for malnutrition diagnosis (1–3). Severe malnutrition can negatively impact cancer patients’ prognosis and therapeutic outcomes, resulting in not only considerable economic losses for the patients but also a waste of valuable medical resources (4). Therefore, it is critical to actively support malnourished oncology patients with nutritional therapy after effective screening and identifying malnutrition status in a scientifically sound manner.

There are a number of clinical nutrition assessment practices with varying degrees of validity, such as the nutrition risk screening 2002 (NRS-2002), mini nutritional assessment–short form (MNA-SF), and malnutrition universal screening tool (MUST), though none of these methods has gained broad universal acceptance (5–8). In response to the urgent need for a global agreement on clinical nutrition evaluation for adults, the Global Leadership Initiative on Malnutrition (GLIM) established the latest criteria for malnutrition diagnosis in 2018 (9). Since then, results from multiple cohorts demonstrated that malnutrition identified by GLIM criteria might be negatively related to both long-term prognosis and short-term in-hospital outcomes in patients with different types of malignancies (10, 11). There still, nevertheless, are inconsistent results among reports. The present meta-analysis aimed to address the value of GLIM-defined malnutrition in predicting survival and clinical outcomes in cancer patients.

## Materials and methods

### Registration

The protocol of this systematic review and meta-analysis has been reported/registered on the PROSPERO database (registration no. CRD42022321094).

### Literature search

The report of this study was conducted according to the guidelines of Preferred Reporting Items for Systematic Reviews and Meta-Analyses (12). Two independent authors (DD Peng and KZ Zong) designed the search strategy and then systematically searched PubMed, Web of Science and Embase databases from their inceptions to September 13, 2022, with a combination of the following key words: “Global Leadership Initiative on Malnutrition” OR “GLIM” AND “cancer” OR “malignancy” OR “carcinoma” AND “survival” OR “mortality” OR “death” AND “outcome” OR “prognosis” OR “complications” OR “readmission” (Supplementary material). Reference lists of pertinent articles were also manually scanned for additional studies. Articles were restricted to human adults (age ≥ 18 years) and written in English.

### Eligibility criteria and study selection

Studies satisfied all the following criteria were enrolled: (1) population: adult patients diagnosed with cancer; (2) exposure: malnutrition by GLIM criteria at baseline; (3) diagnostic criteria: diagnosing malnutrition with at least one phenotypic criterion (weight loss, low body mass index, reduced muscle mass) and one etiologic criterion (reduced food intake or assimilation, inflammation); (4) comparison: cancer patients with a diagnosis of malnutrition by GLIM criteria to those without malnutrition; (5) study type: retrospective or prospective observational studies; (6) outcomes: overall survival (OS), disease-free survival (DFS), postoperative complications or 30-day readmission; (7) hazard ratio (HR) with 95% confidence intervals (CI) for survival outcome and odds ratio (OR) with 95% CI or events in different groups for clinical outcome reported. The study with the most thorough data of different interested outcomes was taken for several papers with participants from the same cohort. The exclusion criteria were as follows: (1) papers published in language other than English; (2) malnutrition diagnosed by tools other than GLIM; (3) did not apply GLIM correctly (4) no interested outcome reported; (5) follow-up duration less than three months if survival-related data were provided. The study selection was carried out separately by two examiners, and inconsistencies were resolved by consultation with a third examiner.

### Data extraction and quality assessment

Two researchers extracted the data independently, and disagreements were solved by communication with a third researcher. The extracted data included: the author’s surname, publication year, the origin of study, study design, sample sizes, age, gender distribution, cancer type, assessment of malnutrition prevalence by GLIM criteria, phenotypic and grading criteria of malnutrition, outcomes measures, length of follow-up. A 9-point Newcastle-Ottawa Scale (NOS) was used for methodological quality evaluation of the included researches (13). Selection, comparability and outcome are the three criteria applied by the NOS to assess quality of studies. According to this scale, low-quality was indicated by 0–3 points, medium-quality by 4–6 points, and good-quality by 7–9 points.

### Data synthesis and analysis

The meta-analysis was conducted using Stata 12.0 (Stata Corporation, College Station, TX). A random-effect meta-analysis model was applied irrespective of heterogeneity. The difference in overall survival between malnourished cancer patients diagnosed by GLIM criteria and well-nourished cancer patients was analyzed by pooling HR with 95% CI. Events in different groups were used to generate OR with 95% CI, or the reported OR was directly extracted. The Cochran Q test and I-squared were used to assess heterogeneity, with I^2^ > 50% or *P* < 0.1 being regarded to indicate significant heterogeneity. Subgroup analysis was conducted based on study design, region, cancer types, mean age of cohort, year of publication, follow-up length, body mass index (BMI) cut-off value for grading or screening tools used to determine the origin of heterogeneity. *P* < 0.05 was used to indicate significant publication bias when using the Egger’s (14) and Begg’s (15) test. Leave-one-out sensitivity analysis was conducted to verify the results’ stability.

## Results

### Search results and study characteristics

**FIGURE 1:**
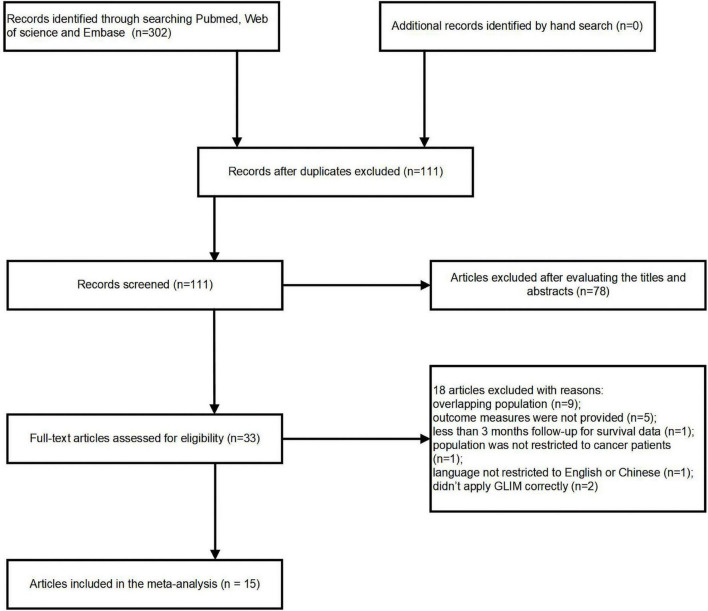
Flow chart depicting the study inclusion and exclusion processes.

302 potentially pertinent articles were found in the original literature review. After duplications were removed, 111 articles remained. Out of these, 33 full-text articles were retrieved for in-depth evaluation, while 78 publications were eliminated after reviewing the titles or abstracts. Applying our inclusion and exclusion criteria resulted in the further exclusion of eighteen publications. Finally, 15 cohort studies (16–30) were pooled into the current analysis (Figure 1). Because Huang (18), Xu (28) and Song (29) reported different outcomes of interest or enrolled patients with different cancer types, they were all included in the present study even though they originated from a same cohort.

Details of the study characteristics and NOS scores were described in Table 1. All enrolled papers were published between 2020 and 2022, including nine from China (18, 20, 21, 24, 26–30), and one each from Japan (19), Australia (22), Greece (16), Turkey (17), Spain (23) and South Korea (25). Four articles (16, 20, 23, 30) were designed as prospective, while eleven (17–19, 21, 22, 24–29) were retrospective. Four articles (31–34) enrolled patients with all types of cancer, three (18, 27, 28) enrolled patients with gastric cancer, three (19, 20, 26) enrolled patients with esophageal cancer, one (16) enrolled patients with gastric/pancreatic/hepatic/colorectal cancer, one (30) enrolled patients with gastric/pancreatic/biliary/colorectal cancer. The remaining three articles included patients diagnosed with hematologic cancer (17), pancreatic cancer (25), or colorectal cancer (29), respectively. Sample size of the cohorts varied from 218 to 3547, with prevalence of malnutrition diagnosed by GLIM differing from 22.0 to 88.0%. The detailed description on diagnostic and grading criteria of malnutrition were present in the Supplementary Tables 1, 2.

**TABLE 1:** Characteristic of the included studies.

References	Region	Cancer types	Study design	Follow-up (month)	Sample size (n)	Number of malnourished (%)	Age (years)	NOS scores
Kakavas et al. (16)	Greece	Gastric/pancreatic/ hepatic/colorectal	Prospective	3	218	72 (22.0)	70.1 ± 13.1	7
Yilmaz et al. (17)	Turkey	Hematologic	Retrospective	12	120	31 (25.8)	53.6 ± 14.1	7
Huang et al. (18)	China	Gastric	Retrospective	72	597	206 (34.5)	65 ± 13	7
Okada et al. (19)	Japan	Esophageal	Retrospective	60	117	51 (43.6)	63.8 ± 11.1	9
Wang et al. (20)	China	Esophageal	Prospective	28	189	143 (75.7)	65.1 ± 7.2	8
Liu et al. (21)	China	All type	Retrospective	/	2388	929 (38.9)	854 (34.3%) over 65	7
Poulter et al. (22)	Australia	All type	Retrospective	1	2679	616 (23.0)	62.7 ± 14.1	7
Sánchez- Torralvo et al. (23)	Spain	All type	Prospective	6	208	183 (88.0)	60.5 ± 12.9	7
Zhang et al. (24)	China	All type	Retrospective	36	3547	2495 (70.3)	59.1 ± 12.8	9
Lee et al. (25)	South Korea	Pancreatic	Retrospective	24.5	228	75 (32.9)	64.7 ± 10.6	7
Yin et al. (26)	China	Esophageal	Retrospective	6	360	120 (33.3)	64.1 ± 7.7	8
Li et al. (27)	China	Gastric	Retrospective	60	994	312 (31.4)	60.0 ± 12.0	7
Xu et al. (28)	China	Gastric	Retrospective	60	895	343 (38.3)	66 (58–73)	8
Song et al. (29)	China	Colorectal	Retrospective	72	918	217 (23.6)	66 ± 17 23.6	7
Tan et al. (30)	China	Gastric/pancreatic/ Biliary/colorectal	Prospective	1	1115	400 (35.9)	62.6 ± 10.8	8

NOS, Newcastle-Ottawa Scale. Selection, comparability and outcome are the three criteria applied by the NOS to assess the quality of studies. 0–3 points indicate low-quality, 4–6 points indicate medium-quality and 7–9 points indicate good-quality.

### Data analysis of survival data

**FIGURE 2:**
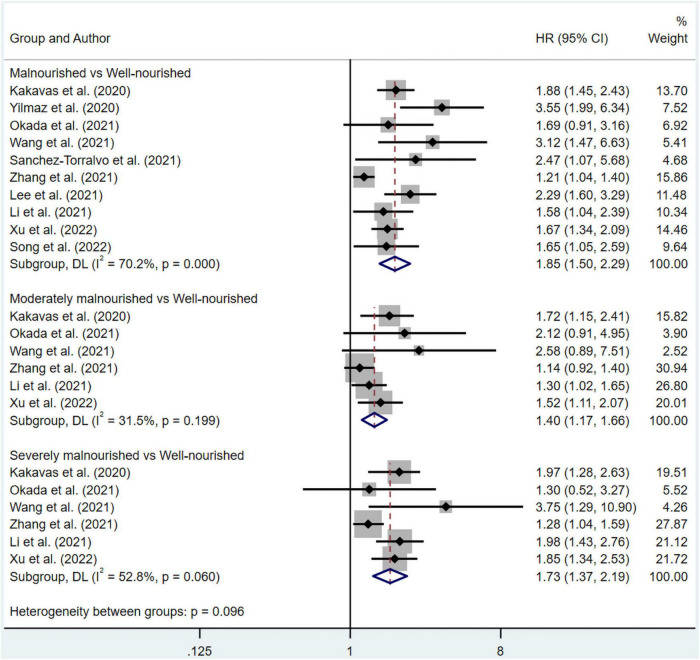
Forest plots showing pooled HR with 95% CI of overall survival under multivariate regression model. X-axis indicated hazard ratio (HR). Weights and between-subgroup heterogeneity test are from random-effects model.

**FIGURE 3:**
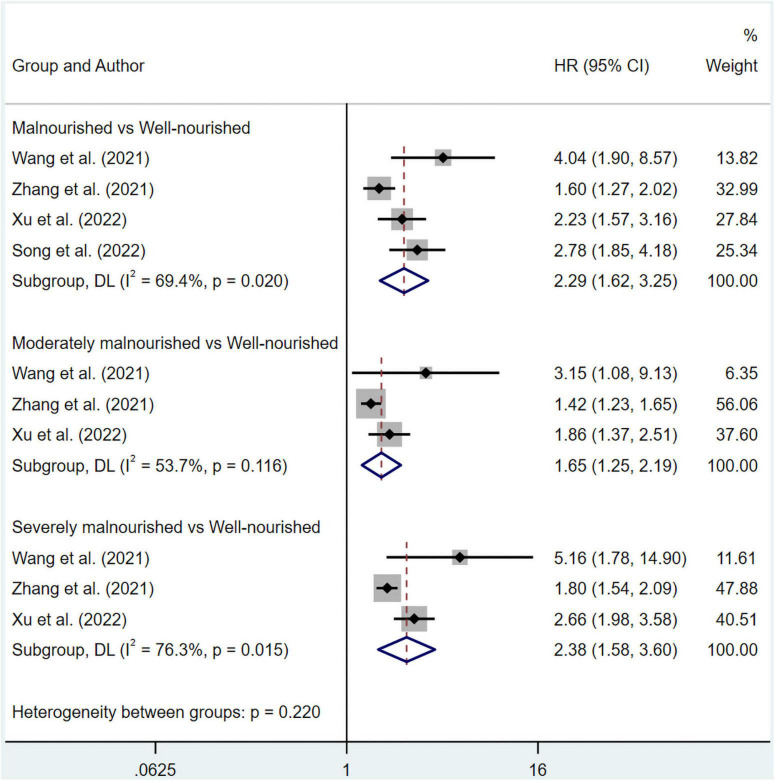
Forest plots showing pooled HR with 95% CI of overall survival under univariate regression model. X-axis indicated hazard ratio (HR). Weights and between-subgroup heterogeneity test are from random-effects model.

The pooled data of overall survival from multivariate and univariate regression analysis were shown. The significant results suggested that patients in the malnourished (HR 1.85, 95% CI 1.50–2.29 for multivariate regression; HR 2.29, 95% CI 1.62–3.25 for univariate regression), moderately malnourished (HR 1.40, 95% CI 1.17–1.66 for multivariate regression; HR 1.65, 95% CI 1.25–2.19 for univariate regression) or severely malnourished groups (HR 1.73, 95% CI 1.37–2.19 for multivariate regression; HR 2.38, 95% CI 1.58–3.60 for univariate regression) all had a worse overall prognosis than those in the well-nourished group under a random effect model (Figures 2, 3). At the same time, significant heterogeneity was found in groups of malnourished (I^2^ = 70.2%, *p* = 0.000 for multivariate regression; I^2^ = 69.4%, *p* = 0.020 for univariate regression), moderately malnourished (I^2^ = 53.7%, *p* = 0.116 for univariate regression) and severely malnourished (I^2^ = 52.8%, *p* = 0.06 for multivariate regression; I^2^ = 76.3%, *p* = 0.015 for univariate regression) when compared with the well-nourished group (Figures 2, 3). Therefore, subgroup analysis was performed (Supplementary Tables 3–7). According to Supplementary Figures 1–9, 11, the stability of the pooled results from all these groups was proved by Leave-one-out sensitivity analysis. The Begg’s and Egger’s tests indicated no likelihood of publication bias in these groups, except Egger’s tests in malnourished *vs* well-nourished group (*p* = 0.04) and moderately malnourished *vs* well-nourished group (*p* = 0.028) showed significance (Supplementary Table 8). After imputing potential missing studies based on trim-and-fill analysis (Supplementary Figures 2–10, 12), no significant alteration in HR value and its’ range was observed for all of the aforementioned groups (Supplementary Table 8).

**FIGURE 4:**
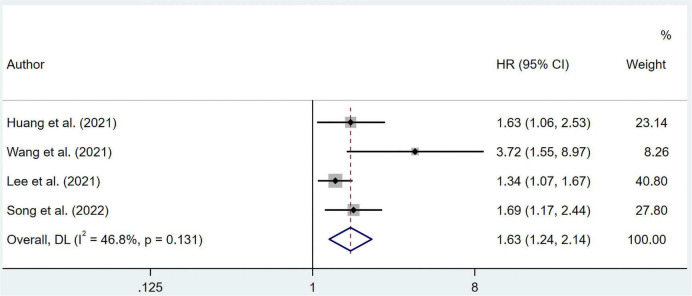
Forest plots showing pooled HR with 95% CI of disease-free survival under multivariate regression model. X-axis indicated hazard ratio (HR). Weights are from random-effects model.

As for DFS, the combined HR from multivariate regression was 1.63 (95% CI 1.24–2.14) without apparent heterogeneity (I^2^ = 46.8%, *p* = 0.131) (Figure 4). The likelihood of publication bias was revealed by the Egger’s test (*p* = 0.025), but not by the Begg’s tests (*p* = 0.308). The pooled HR for DFS was 1.419 (95% CI 1.076–1.873) after imputing 2 possible misses (Supplementary Figure 14).

### Data analysis of clinical outcomes data

**FIGURE 5:**
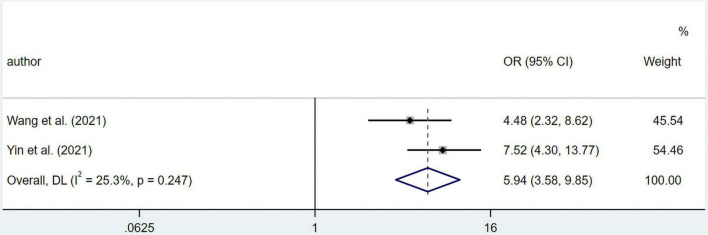
Forest plots showing pooled HR with 95% CI of overall complications under multivariate regression model. X-axis indicated odds ratio (OR). Weights are from random-effects model.

**FIGURE 6:**
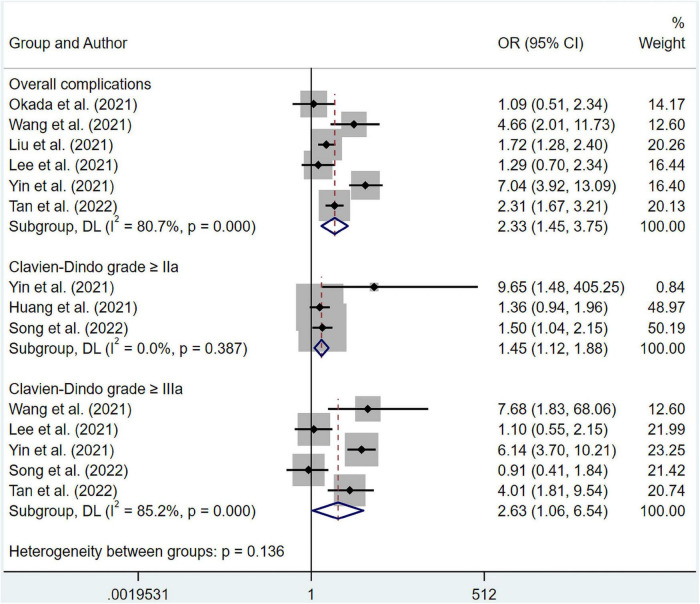
Forest plots showing pooled HR with 95% CI of postoperative complications under univariate regression model. X-axis indicated odds ratio (OR). Weights and between-subgroup heterogeneity test are from random-effects model.

The results suggested that malnourished patients experienced higher risk in overall complications compared to well-nourished patients (OR 5.94; 95% CI 3.58–9.85; I^2^ = 25.3%; *p* = 0.247) based on multivariate regression (Figure 5). Meanwhile, pooled OR from univariate regression, which were 2.33 (95% CI 1.45–3.75; I^2^ = 80.7%; *p* = 0.000) for overall survival, 1.45 (95% CI 1.12–1.88; I^2^ = 0%; *p* = 0.387) for complications ≥ Clavien-Dindo grade IIa and 2.63 (95% CI 1.06–6.54; I^2^ = 85.2%; *p* = 0.000) for complications ≥ Clavien-Dindo grade IIIa, indicated a similar conclusion (Figure 6). Subgroup analysis was performed for groups of overall complications and complications ≥ Clavien-Dindo grade IIIa (Supplementary Tables 9, 10). Notably, change in statistical significance was observed in sensitivity analysis for groups of complications ≥ Clavien-Dindo grade IIa and ≥ Clavien-Dindo grade IIIa (Supplementary Figures 15, 17, 19). No statistical significance was reported in both Begg’s and Egger’s tests (Supplementary Table 11). The pooled OR did not change significantly after imputing potential missing publications (Supplementary Figures 16, 18, 20).

Two papers by Poulter et al. and Tan et al. (22, 30) reported the connection between GLIM-defined malnutrition and 30-day readmission, with OR = 1.78 (95% CI 1.34–2.35) under multivariate model for one and OR = 0.71 (0.36–1.34) under univariate model for another. As for 30-day mortality, only one paper reported (23), with OR = 2.50 (95% CI 1.44–4.35) under multivariate model.

## Discussion

The present Meta-analysis provided a multidimensional insight into the predictive role of GLIM-defined malnutrition on the survival of cancer patients, and reported for the first time its prognostic impact on clinical outcomes for cancer patients undergoing surgery. Specifically, cancer patients with different grades of malnutrition diagnosed by GLIM criteria had worse overall survival and DFS than those with good nutrition status, also facing a higher risk of postoperative overall complications, complications ≥ Clavien-Dindo grade IIa and ≥ Clavien-Dindo grade IIIa.

Malnutrition is prevalent in adult cancer patients, with its severity being influenced by a variety of factors, including cancer stage, tumor site, age, and underlying illnesses. Moreover, ongoing malnutrition progresses into cachexia, a deteriorated condition characterized by uncontrollable loss of body composition and compromised physical function (35). Since studies have given evidence on the negative impacts of malnutrition on cancer patients in numerous aspects, a nutritional assessment should be performed for all cancer patients in a whole-course manner (36). However, depending on the methods adopted, malnutrition prevalence varies (37). The GLIM criteria were introduced to achieve a worldwide consensus on the diagnostic standards for malnutrition in 2018. The criteria required being validated for applicability in different clinical settings, though, as it was proposed on the basis of the collective expertise of specialists (9).

Before an in-depth interpretation of the results, some important issues must be noted. In order to better spread the criteria, GLIM recommends a variety of practical tools for application by medical institutions in different regions. For example, screening by any of the nutritional risk scales currently in clinical use prior to the final diagnosis of malnutrition was recommended by GLIM. However, it was not specified which screening scale was used in some of the included literature, as differences in the efficacy of various scales may have some impact on the risk population included. In addition, reduced muscle mass, one of the three phenotypic criteria for malnutrition, can be diagnosed by a variety of tools, including skeletal muscle index (SMI) and calf circumference (CC). No uniformity in the means of detection was achieved for enrolled articles. By the same token, this could potentially affect the population of malnourished patients eventually diagnosed by GLIM. Individuals with cancer diagnosed with GLIM defined malnutrition conferred a worse overall survival compared to those without, according to our results of pooled adjusted or unadjusted HR values for survival data. This is in line with the prevailing perception that specialized nutritional therapy is beneficial for survival of malnourished patients (38, 39). We determined through subgroup analysis that heterogeneity was most likely attributed to cancer types. In fact, given that Asian population made up the majority of this meta-analysis, there might be some heterogeneity generated by the utilization of various BMI standards. The GLIM recommended BMI < 18.5 kg/m^2^ for patients younger than 70 and < 20 kg/m^2^ for patients over 70 as the cut-off reference of malnutrition grading for Asian populations (9). However, Maeda et al. from Japan proposed that the preferred BMI cutoff value for younger adults should fall to 17.0 kg/m^2^, while value for older adults to 17.8 kg/m^2^ (40). We noticed that three articles from Asia adopted this range (27, 28, 30) (Supplementary Table 2), and subgroup analysis revealed a reduced heterogeneity in both subgroups chose different cut-off values (Supplementary Table 6). Therefore, in-depth investigations on this topic, which enroll patients with one certain type of cancer, are needed to encourage high-quality meta-analysis that focuses on the effect of GLIM-defined malnutrition on specific type of cancer in the future. As well, the BMI criteria for Asian patients require further validation to reach a scientific and unified standard.

Although previous meta-analyses have identified the negative impact of malnutrition on postoperative complications in surgical patients (41, 42), Torbahn et al. reported malnutrition screened by MNA showed no advantage in predicting postoperative complications and treating toxicity in patients with cancer (31). To shed light on this issue, we conducted meta-analysis on the impact of GLIM-defined malnutrition on postoperative complications in cancer patients for the first time. Our pooled analysis of multivariate adjusted OR values revealed that patients with esophageal cancer are 5.94 times more likely to experience postoperative complications if they are malnourished, as defined by GLIM. Similarly, the integrated results of unadjusted OR values suggested that malnourished patients with various cancers faced higher risk of postoperative complications. The subgroup analysis was unable to determine the source of the heterogeneity in the overall complications group under univariate regression, indicating that heterogeneity was precipitated by a combination of multiple parameters. But we found age differences and cancer types as possible contributors of heterogeneity in result of complications ≥ Clavien-Dindo grade IIIa (Supplementary Table 10). Notably, the pooled results of the remaining studies became insignificant after a few of the literature was excluded (Supplementary Figures 16, 18). Hence regretfully, the results of these two groups lacked stability, which might be mainly due to a limited number of included studies and different cancer types in each study. Therefore, more high-quality studies are in urge to provide additional data in the future.

Few other limitations need to be addressed in this meta-analysis. Analysis of 30-day readmission and mortality was inadequate, limited by the data provided in the included literature. Additionally, most included papers were in retrospective design, which might influence selection bias.

Altogether, with thoughtful and rigorous design aims advancing the global agreement on malnutrition diagnosis, the criteria defined by the GLIM should be recommended as it contributes to an effective screening of patients for malnutrition. Not only predicts poor survival and clinical prognosis in cancer patients based on our results, but GLIM-defined malnutrition also possesses the equivalent efficacy in non-oncology patients in various clinical settings, according to many reports (32–34). Hopefully, appropriate nutritional support for these patients could considerably improve their overall quality of life.

## Conclusion

Global Leadership Initiative on Malnutrition-defined malnutrition holds value in predicting survival for cancer patients and clinical outcomes, including postoperative complications, 30-day mortality and 30-day readmission for those who receive surgery to remove malignancies.

## Data availability statement

The original contributions presented in the study are included in the article/Supplementary material, further inquiries can be directed to the corresponding author.

## Author contributions

DP: design, data curation, formal analysis, investigation, and writing original draft. KZ: data curation, formal analysis, and visualization. ZH: methodology and software. HY: execution. TM: visualization. ZW: reviewing and supervision. All authors contributed to the article and approved the submitted version.
