# Supplementary material for: Malnutrition diagnosed by the Global Leadership Initiative on Malnutrition criteria predicting survival and clinical outcomes of patients with cancer: A systematic review and meta-analysis
Source: Front Nutr. 2022 Dec 6;9:1053165. doi: 10.3389/fnut.2022.1053165 (PMC9763567; doi:10.3389/fnut.2022.1053165)
Supplement: Supplementary file 1 [file Table_1.DOCX]

Supplementary Material

# Search Strategy

Pubmed:

((“Global Leadership Initiative on Malnutrition” OR “GLIM”) AND ("cancer" OR "malignancy" OR "carcinoma")) AND (("survival" OR "mortality" OR "death") OR ("outcome" OR "prognosis" OR "complications" OR "readmission"))

Web of science

| #1 | 'global leadership initiative on malnutrition' (Topic) or GLIM (Topic) |
| --- | --- |
| #2 | cancer (Topic) or malignancy (Topic) or carcinoma (Topic) |
| #3 | survival (Topic) or mortality (Topic) or death (Topic) or outcome (Topic) or prognosis (Topic) or complication* (Topic) or readmission(Topic) |
| #4 | (#1 AND #2) AND #3 |

Embase

| #1 | 'global leadership initiative on malnutrition':ti,ab,kw OR GLIM:ti,ab,kw |
| --- | --- |
| #2 | cancer:ti,ab,kw OR malignancy:ti,ab,kw OR carcinoma:ti,ab,kw |
| #3 | survival:ti,ab,kw OR mortality:ti,ab,kw OR death:ti,ab,kw OR outcome*:ti,ab,kw OR prognosis*:ti,ab,kw OR complications*:ti,ab,kw OR readmission:ti,ab,kw |
| #4 | #1 AND #2 |
| #5 | #3 AND #4 |

**Supplementary Tables**

Supplemental Table 1 Description on phenotypic criteria of malnutrition and screening tool used.

| Author/year | Phenotypic criteria | | | Screening tool |
| --- | --- | --- | --- | --- |
|  | Weight loss | Low BMI (kg/m^2^) | Reduced muscle mass |  |
| Kakavas et al. (2020) | > 5% within past 6 month | < 20 if < 70 years, or <22 if >70 years. | Calf circumference < 31cm | Not specified |
| Yilmaz et al. (2020) | > 5% within past 6 months or > 10% beyond 6 months | < 20 if < 70 years, or <22 if >70 years. | Fat-free mass index < 17kg/m^2^ for male or 15kg/m^2^ for female | NRS-2002 |
| Huang et al. (2021) | > 5% within past 6 months or > 10% beyond 6 months | < 18.5 if < 70 years, or < 20 if > 70 years | Skeletal muscle index at the cross-section of third lumbar vertebra < 40.8cm^2^/m^2^ for male or < 34.9cm^2^/m^2^ for female | NRS-2002 |
| Okada et al. (2021) | 5%-10% within past 6 months | < 18.5 if < 70 years, or < 20 if > 70 years | Skeletal muscle index < 7.0kg/m^2^ for male or < 5.7kg/m^2^ for female | Not specified |
| Wang et al. (2021) | > 5% within past 6 months or > 10% beyond 6 months | < 20 if < 70 years, or <22 if >70 years. | Skeletal muscle index < 9.87kg/m^2^ for men and < 7.15kg/m^2^ for women | MUST, NRS 2002, MNA-SF, PNI, GNRI |
| Liu et al. (2021) | > 5% within past 6 months or > 10% beyond 6 months | < 18.5 if < 70 years, or < 20 if > 70 years | Calf circumference < 34cm and handgrip strength <28kg for male, calf circumference < 33cm and handgrip strength <18kg for female | NRS-2002 |
| Poulter et al. (2021) | >5% within past 3 months or >10% beyond 4 months | < 20 if < 70 years, or <22 if >70 years. | > 4 muscle sites rated as mild/moderate/severe deficit | MST |
| Sanchez-Torralvo et al. (2021) | > 5% within past 6 months or > 10% beyond 6 months | < 20 if < 70 years, or <22 if >70 years. | Skeletal muscle index at the cross-section of third lumbar vertebra <43cm^2^/m^2^ for male with BMI < 25.0kg/m^2^ or < 53cm^2^/m^2^ for male with BMI > 25.0 kg/m^2^. < 41cm^2^/m^2^ for female | Not specified |
| Zhang et al. (2021) | > 5% within past 6 months or > 10% beyond 6 months | < 20 if < 70 years, or <22 if >70 years.  Asia: < 18.5 if < 70 years, or < 20 if > 70 years | Calf circumference < 30cm for male and <29cm for female; handgrip strength per weight < 0.3305 for male and < 0.2144 for female; mid-arm muscle circumference < 18.66cm for male and < 17.06cm for female. | Not specified |
| Lee et al. (2021) | > 5% within past 6 months or > 10% beyond 6 months | < 18.5 if < 70 years, or < 20 if > 70 years | By validated body composition measuring techniques | Not specified |
| Yin et al. (2021) | > 5% within past 6 months or > 10% beyond 6 months | < 18.5 if < 70 years, or < 20 if > 70 years | Calf circumference < 30cm for male and < 29cm for female | Not specified |
| Li et al. (2021) | > 5% within past 6 months or > 10% beyond 6 months | < 18.5 if < 70 years, or < 20 if > 70 years | Skeletal muscle index ≤ 40.8cm^2^/m^2^ for men and ≤ 34.9cm^2^/m^2^ for women | NRS-2002 |
| Xu et al. (2022) | > 5% within past 6 months or > 10% beyond 6 months | < 18.5 if < 70 years, or < 20 if > 70 years | Skeletal muscle index ≤ 40.8cm^2^/m^2^ for men and ≤ 34.9cm^2^/m^2^ for women | Not specified |
| Song et al. (2022) | > 5% within past 6 months or > 10% beyond 6 months | < 18.5 if < 70 years, or < 20 if > 70 years | Handgrip strength < 19kg for male or < 10kg for female | NRS-2002 |
| Tan et al. (2022) | 5%-10% within past 6 months | < 18.5 if < 70 years, or < 20 if > 70 years | 36.17 ≤ Skeletal muscle index ≤ 43.13 for male and 29.89 ≤ Skeletal muscle index ≤ 37.81 for female | NRS-2002, MNA-SF, MUST, PONS |

Supplemental Table 2 Description on grading criteria of malnutrition.

| Author/year | Phenotypic criteria for severe malnutrition | | |
| --- | --- | --- | --- |
|  | Weight loss | Low BMI (kg/m^2^) | Reduced muscle mass |
| Kakavas et al. (2020) | > 10% within past 6 months | < 18.5 if < 70 years, or < 20 if > 70 years | Calf circumference < 31cm |
| Yilmaz et al. (2020) | > 10% within past 6 months or > 20% beyond 6 months | < 18.5 if < 70 years, or < 20 if > 70 years | Severe deficit by validated body composition measuring techniques |
| Huang et al. (2021) | / | / | / |
| Okada et al. (2021) | > 10% in 6 months | Not applicable | Skeletal muscle index < 6.1kg/m^2^ for male or < 5.0kg/m^2^ for female |
| Wang et al. (2021) | > 10% within past 6 months or > 20% beyond 6 months | < 18.5 if < 70 years, or < 20 if > 70 years | Skeletal muscle index < 8.87kg/m^2^ for men and < 6.42kg/m^2^ for women |
| Liu et al. (2021) | > 10% within past 6 months or > 20% beyond 6 months | < 17.0 if < 70 years, or < 17.8 if > 70 years | / |
| Poulter et al. (2021) | / | / | / |
| Sanchez-Torralvo et al. (2021) | / | / | / |
| Zhang et al. (2021) | > 10% within past 6 months or > 20% beyond 6 months | Not applicable | Calf circumference < 27.5cm for male and <27cm for female; handgrip strength per weight < 0.2267 for male and < 0.1375 for female; mid-arm muscle circumference < 16.49cm for male and < 15.08cm for female. |
| Lee et al. (2021) | / | / | / |
| Yin et al. (2021) | / | < 17.0 if < 70 years, or < 17.8 if > 70 years | / |
| Li et al. (2021) | > 10% within past 6 months or > 20% beyond 6 months | < 17.0 if < 70 years, or < 17.8 if > 70 years | Skeletal muscle index ≤ 34.5cm^2^/m^2^ for men and ≤ 28.9cm^2^/m^2^ for women |
| Xu et al. (2022) | > 10% within past 6 months or > 20% beyond 6 months | < 17.0 if < 70 years, or < 17.8 if > 70 years | Skeletal muscle index ≤ 34.5cm^2^/m^2^ for men and ≤ 28.9cm^2^/m^2^ for women |
| Song et al. (2022) | / | / | / |
| Tan et al. (2022) | > 10% in 6 months | < 17.0 if < 70 years, or < 17.8 if > 70 years | Skeletal muscle index < 36.17cm^2^/m^2^ for men and < 29.89cm^2^/m^2^ for women |

| Supplemental Table 3 Subgroup analysis of overall survival under multivariate regression model (malnourished vs well-nourished). | | | | | | | |
| --- | --- | --- | --- | --- | --- | --- | --- |
| Subgroup | No. of articles | pooled HR | | 95% CI | I^2^ (%) | P | P-interaction |
| Study design |  | |  |  |  |  | 0.424 |
| Prospective | 3 | | 2.02 | 1.6-2.55 | 0 | 0.407 |  |
| Retrospective | 7 | | 1.75 | 1.37-2.25 | 74.0 | 0.001 |  |
| Region |  | |  |  |  |  | 0.784 |
| Asia | 8 | | 1.83 | 1.43-2.35 | 73.8 | 0 |  |
| Others | 2 | | 1.93 | 1.50-2.46 | 0 | 0.540 |  |
| Cancer types |  | |  |  |  |  | 0.126 |
| All | 2 | | 1.53 | 0.79-2.95 | 63.3 | 0.099 |  |
| Abdominal digestive | 5 | | 1.79 | 1.56-2.06 | 0 | 0.595 |  |
| Esophageal | 2 | | 2.21 | 1.22-4.01 | 33.9 | 0.219 |  |
| Hematologic | 1 | | 3.55 | 1.99-6.34 | 0 | / |  |
| Follow-up length |  | |  |  |  |  | 0.158 |
| ≤ 24 months | 3 | | 2.38 | 1.56-3.64 | 50.0 | 0.135 |  |
| ＞24 months | 7 | | 1.68 | 1.34-2.11 | 66.8 | 0.006 |  |
| Screening tool specified |  | |  |  |  |  | 0.320 |
| Yes | 4 | | 2.18 | 1.45-3.27 | 54.7 | 0.071 |  |
| No | 6 | | 1.71 | 1.34-2.18 | 73.2 | 0.002 |  |

| Supplemental Table 4 Subgroup analysis of overall survival under univariate regression model (malnourished vs well-nourished). | | | | | | |
| --- | --- | --- | --- | --- | --- | --- |
| Subgroup | No. of articles | pooled HR | 95% CI | I^2^ (%) | P | P-interaction |
| Study design |  |  |  |  |  | 0.114 |
| Prospective | 1 | 4.04 | 1.90-8.57 | 0 | / |  |
| Retrospective | 4 | 2.08 | 1.49-2.90 | 68.1 | 0.044 |  |
| Cancer types |  |  |  |  |  | **0.010** |
| All | 2 | 1.60 | 1.27-2.02 | 0 | / |  |
| Abdominal digestive | 2 | 2.45 | 1.88-3.19 | 0 | 0.419 |  |
| Esophageal | 1 | 4.04 | 1.9-8.57 | 0 | / |  |
| Screening tool specified |  |  |  |  |  | **0.042** |
| Yes | 2 | 1.84 | 1.33-2.54 | 58.4 | 0.121 |  |
| No | 2 | 3.03 | 2.12-4.33 | 0 | 0.393 |  |
|  |  |  |  |  |  |  |

| Supplemental Table 5 Subgroup analysis of overall survival under univariate regression model (moderately malnourished vs well-nourished). | | | | | | |
| --- | --- | --- | --- | --- | --- | --- |
| Subgroup | No. of articles | pooled HR | 95% CI | I^2^ (%) | P | P-interaction |
| Study design |  |  |  |  |  | 0.214 |
| Prospective | 1 | 3.15 | 1.08-9.16 | 0 | / |  |
| Retrospective | 2 | 1.57 | 1.22-2.03 | 59.5 | 0.116 |  |
| Time of publication |  |  |  |  |  | 0.889 |
| 2021 | 2 | 1.76 | 0.88-3.53 | 52.4 | 0.147 |  |
| 2022 | 1 | 1.86 | 1.37-2.52 | 0 | / |  |
|  |  |  |  |  |  |  |
|  |  |  |  |  |  |  |

| Supplemental Table 6 Subgroup analysis of overall survival under multivariate regression model (severely malnourished vs well-nourished). | | | | | | | |
| --- | --- | --- | --- | --- | --- | --- | --- |
| Subgroup | No. of articles | pooled HR | | 95% CI | I^2^ (%) | P | P-interaction |
| Study design |  |  |  | |  |  | 0.252 |
| Prospective | 2 | 2.22 | | 1.36-3.61 | 20.3 | 0.263 |  |
| Retrospective | 4 | 1.61 | | 1.24-2.08 | 54.6 | 0.086 |  |
| Region |  |  | |  |  |  | 0.512 |
| Asia | 5 | 1.69 | | 1.28-2.24 | 56.7 | 0.055 |  |
| Others | 1 | 1.97 | | 1.37-2.82 | 0 | / |  |
| Cancer types |  |  | |  |  |  | **0.017** |
| All | 1 | 1.28 | | 1.04-1.58 | 0 | / |  |
| Abdominal digestive | 3 | 1.93 | | 1.59-2.34 | 0 | 0.948 |  |
| Esophageal | 2 | 2.13 | | 0.76-6 | 54 | 0.14 |  |
| Follow-up length |  |  | |  |  |  | 0.512 |
| ≤ 24 months | 1 | 1.97 | | 1.37-2.28 | 0 | / |  |
| ＞24 months | 5 | 1.69 | | 1.28-2.24 | 56.7 | 0.055 |  |
| BMI cut-off for grading |  |  | |  |  |  | 0.593 |
| < 17kg/m2 | 2 | 1.91 | | 1.52-2.4 | 0 | 0.754 |  |
| < 18.5kg/m2 | 2 | 2.22 | | 1.36-3.61 | 20.3 | 0.263 |  |
| Screening tool specified |  |  | |  |  |  | 0.228 |
| Yes | 4 | 1.59 | | 1.23-2.05 | 50.7 | 0.107 |  |
| No | 2 | 2.21 | | 1.38-3.53 | 19.9 | 0.264 |  |

| Supplemental Table 7 Subgroup analysis of overall survival under univariate regression model (severely malnourished vs well-nourished). | | | | | | |
| --- | --- | --- | --- | --- | --- | --- |
| Subgroup | No. of articles | pooled HR | 95% CI | I^2^ (%) | P | P-interaction |
| Study design |  |  |  |  |  | 0.127 |
| Prospective | 1 | 5.16 | 1.78-14.93 | 0 | / |  |
| Retrospective | 2 | 2.14 | 1.46-3.13 | 81.1 | 0.022 |  |
| Time of publication |  |  |  |  |  | 0.999 |
| 2021 | 2 | 2.66 | 0.98-7.20 | 73.0 | 0.054 |  |
| 2022 | 1 | 2.66 | 1.98-3.58 | 0 | / |  |

| Supplemental Table 8 Analysis of publication bias for overall survival | | | | | | |
| --- | --- | --- | --- | --- | --- | --- |
| model | Groups |  |  | Trim-and-fill analysis | | |
|  |  | Begg's test | Egger's test | No. of imputation | HR | 95% CI |
| Multivariate regression | |  |  |  |  |  |
|  | Malnourished *vs* Well-nourished | 0.283 | **0.01** | 6 | 1.410 | 1.144-1.737 |
|  | Moderately malnourished *vs* Well-nourished | 0.060 | **0.028** | 3 | 1.280 | 1.065-1.540 |
|  | Severely malnourished *vs* Well-nourished | 0.260 | 0.235 | 1 | 1.670 | 1.308-2.132 |
| Univariate regression |  |  |  |  |  |  |
|  | Malnourished *vs* Well-nourished | 0.089 | 0.050 | 2 | 1.811 | 1.273-2.578 |
|  | Moderately malnourished *vs* Well-nourished | 1.000 | 0.222 | 2 | 1.420 | 1.086-1.857 |
|  | Severely malnourished *vs* Well-nourished | 1.000 | 0.273 | 2 | 1.800 | 1.231-2.633 |

| Supplemental Table 9 Subgroup analysis of overall complications under univariate regression model (malnourished vs well-nourished). | | | | | | |
| --- | --- | --- | --- | --- | --- | --- |
| Subgroup | No. of articles | pooled HR | 95% CI | I^2^ (%) | P | P-interaction |
| Study design |  |  |  |  |  | 0.486 |
| Prospective | 2 | 2.9 | 1.52-5.52 | 53.3 | 0.143 |  |
| Retrospective | 4 | 2.05 | 0.98-4.28 | 86.5 | 0 |  |
| Age |  |  |  |  |  | 0.202 |
| ≤65 | 4 | 2.23 | 1.09-4.55 | 85.4 | 0 |  |
| ＞65 | 1 | 4.66 | 1.93-11.27 | 0 | / |  |
| Cancer types |  |  |  |  |  | 0.556 |
| All | 1 | 1.72 | 1.25-2.35 | 0 | / |  |
| Abdominal digestive | 2 | 1.82 | 1.04-3.20 | 64.5 | 0.093 |  |
| Esophageal | 3 | 3.33 | 1.05-10.56 | 86.2 | 0.001 |  |
| Time of publication |  |  |  |  |  | 0.958 |
| 2021 | 5 | 2.36 | 1.21-4.57 | 84.4 | 0 |  |
| 2022 | 1 | 2.31 | 1.67-3.20 | 0 | / |  |
| Screening tool specified |  |  |  |  |  | 0.950 |
| Yes | 3 | 2.17 | 0.65-7.23 | 90.2 | 0 |  |
| No | 3 | 2.26 | 1.52-3.35 | 60.3 | 0.081 |  |

| Supplemental Table 10 Subgroup analysis of complications ≥ Clavien-Dindo grade IIIa under univariate regression model (malnourished vs well-nourished). | | | | | | |
| --- | --- | --- | --- | --- | --- | --- |
| Subgroup | No. of articles | pooled HR | 95% CI | I^2^ (%) | P | P-interaction |
| Study design |  |  |  |  |  |  |
| Prospective | 2 | 4.49 | 2.11-9.56 | 0 | 0.522 | 0.252 |
| Retrospective | 3 | 1.87 | 0.51-6.84 | 91.9 | 0 |  |
| Age |  |  |  |  |  | **0** |
| ≤65 | 3 | 5.57 | 3.65-8.49 | 0 | 0.65 |  |
| ＞65 | 2 | 1.01 | 0.61-1.67 | 0 | 0.707 |  |
| Cancer types |  |  |  |  |  | **0.006** |
| Abdominal digestive | 3 | 1.55 | 0.65-3.68 | 74.7 | 0.019 |  |
| Esophageal | 2 | 6.24 | 3.82-10.18 | 0 | 0.815 |  |
| Time of publication |  |  |  |  |  |  |
| 2021 | 3 | 3.41 | 0.87-13.31 | 87.9 | 0 | 0.560 |
| 2022 | 2 | 1.88 | 0.44-8.10 | 85.2 | 0.009 |  |
| Screening tool specified |  |  |  |  |  | 0.994 |
| Yes | 3 | 2.62 | 0.74-9.23 | 78.0 | 0.011 |  |
| No | 2 | 2.64 | 0.49-14.22 | 93.6 | 0 |  |

| Supplemental Table 11 Analysis of publication bias for postoperative complications. | | | | | | |
| --- | --- | --- | --- | --- | --- | --- |
| model | Groups |  |  | Trim-and-fill analysis | | |
|  |  | Begg's test | Egger's test | No. of imputation | OR | 95% CI |
| Univariate regression |  |  |  |  |  |  |
|  | Overall complications | 1 | 0.647 | 1 | 1.888 | 1.100-3.239 |
|  | Complications Clavien-Dindo grade ≥ IIa | 1 | 0.177 | 2 | 1.427 | 1.028-1.982 |
|  | Complications Clavien-Dindo grade ≥ IIIa | 1 | 0.847 | 0 | / | / |

**Supplementary Figures**


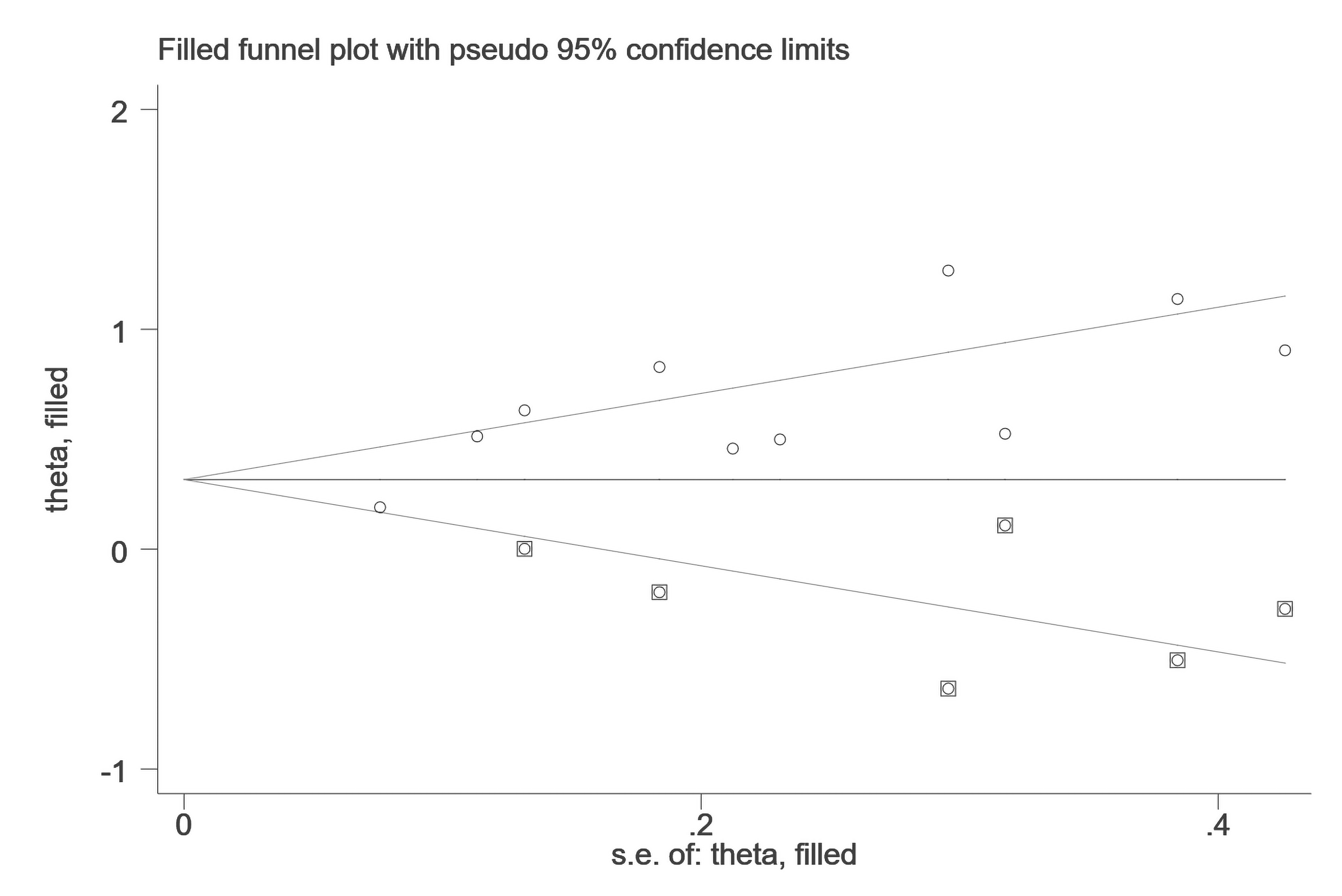

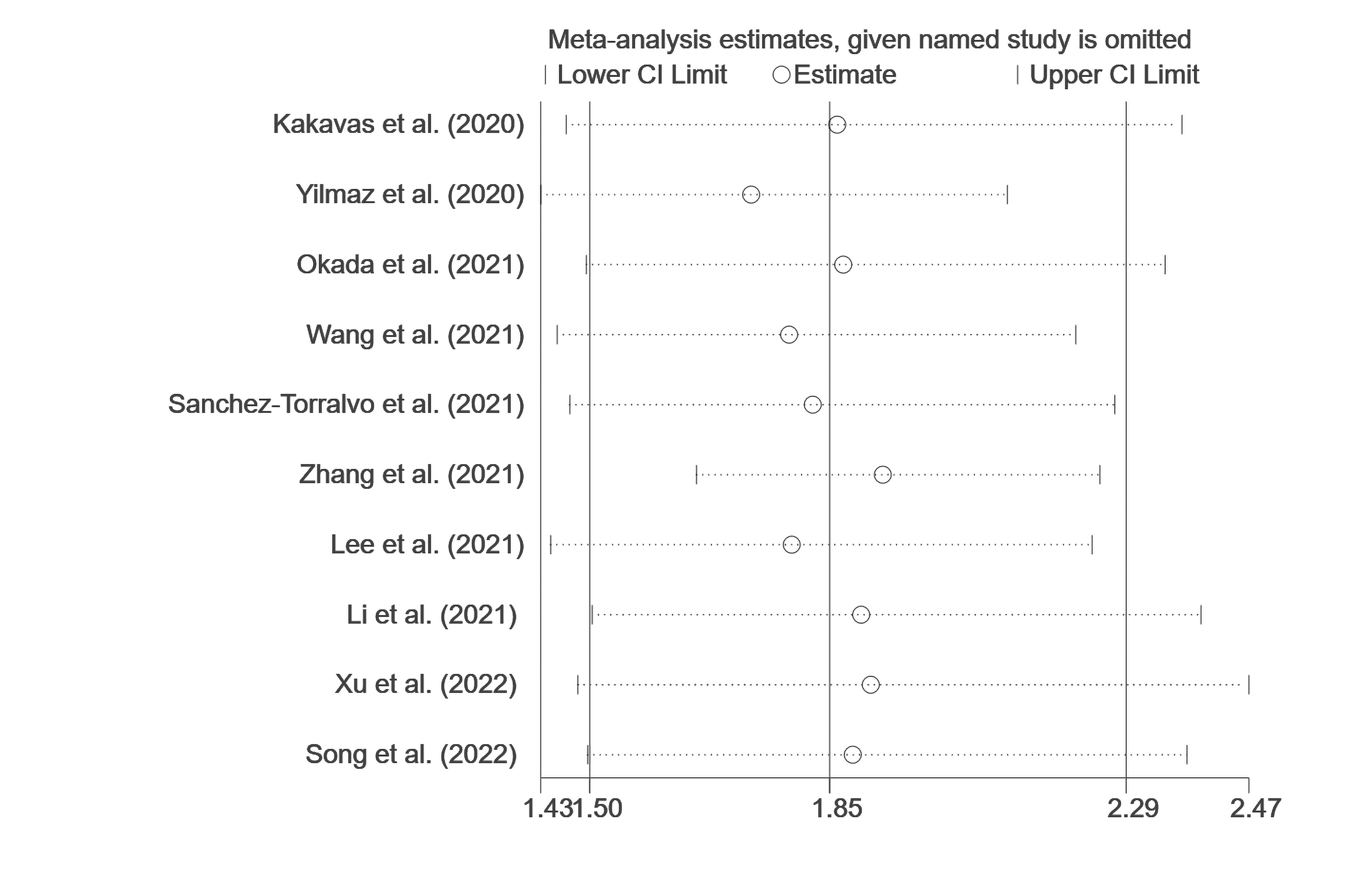


Supplemental Figure 1 Sensitive analysis for pooled result of overall survival under multivariate regression model (malnourished vs well-nourished). The circles represent the pooled results after removing individual study. “|” represent the lower and upper 95%CI limit.

Supplemental Figure 2 Funnel plot showing enrolled studies of overall survival under multivariate regression model (malnourished vs well-nourished). The circles alone are real studies and the circles enclosed in boxes are "imputed" studies.


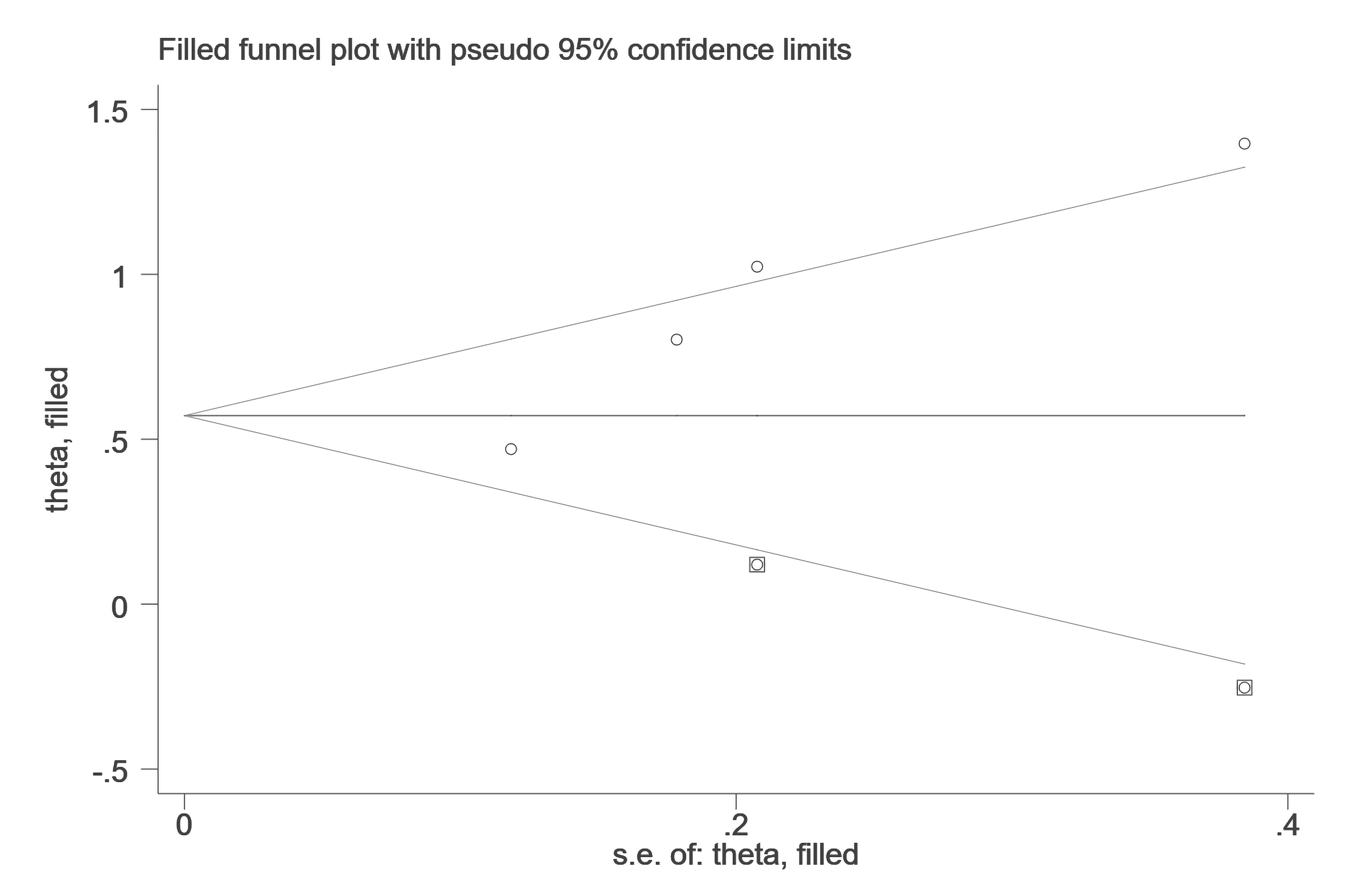

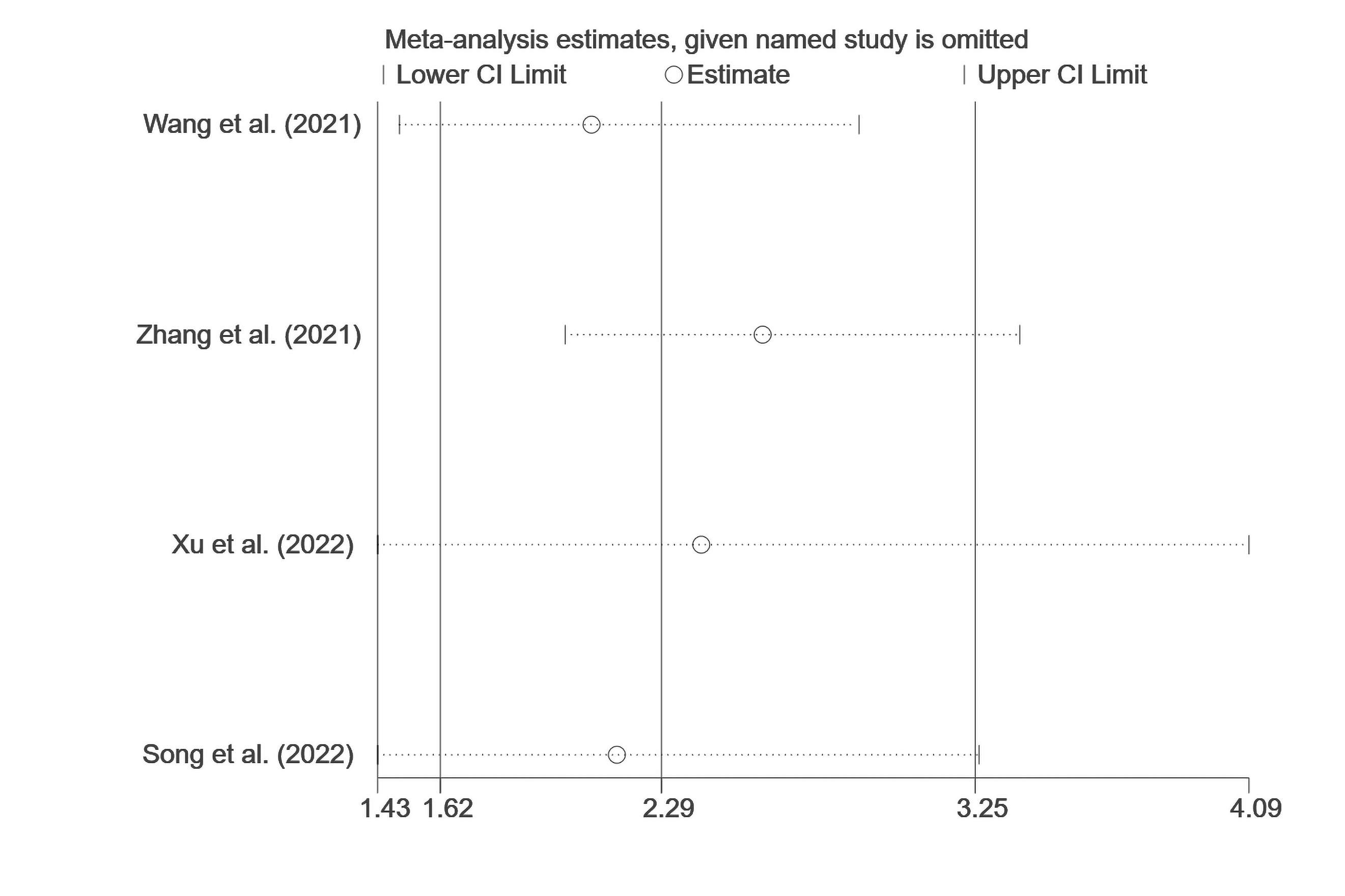


Supplemental Figure 3 Sensitive analysis for pooled result of overall survival under univariate regression model (malnourished vs well-nourished). The circles represent the pooled results after removing individual study. “|” represent the lower and upper 95%CI limit.

Supplemental Figure 4 Funnel plot showing enrolled studies of overall survival under univariate regression model (malnourished vs well-nourished). The circles alone are real studies and the circles enclosed in boxes are "imputed" studies.


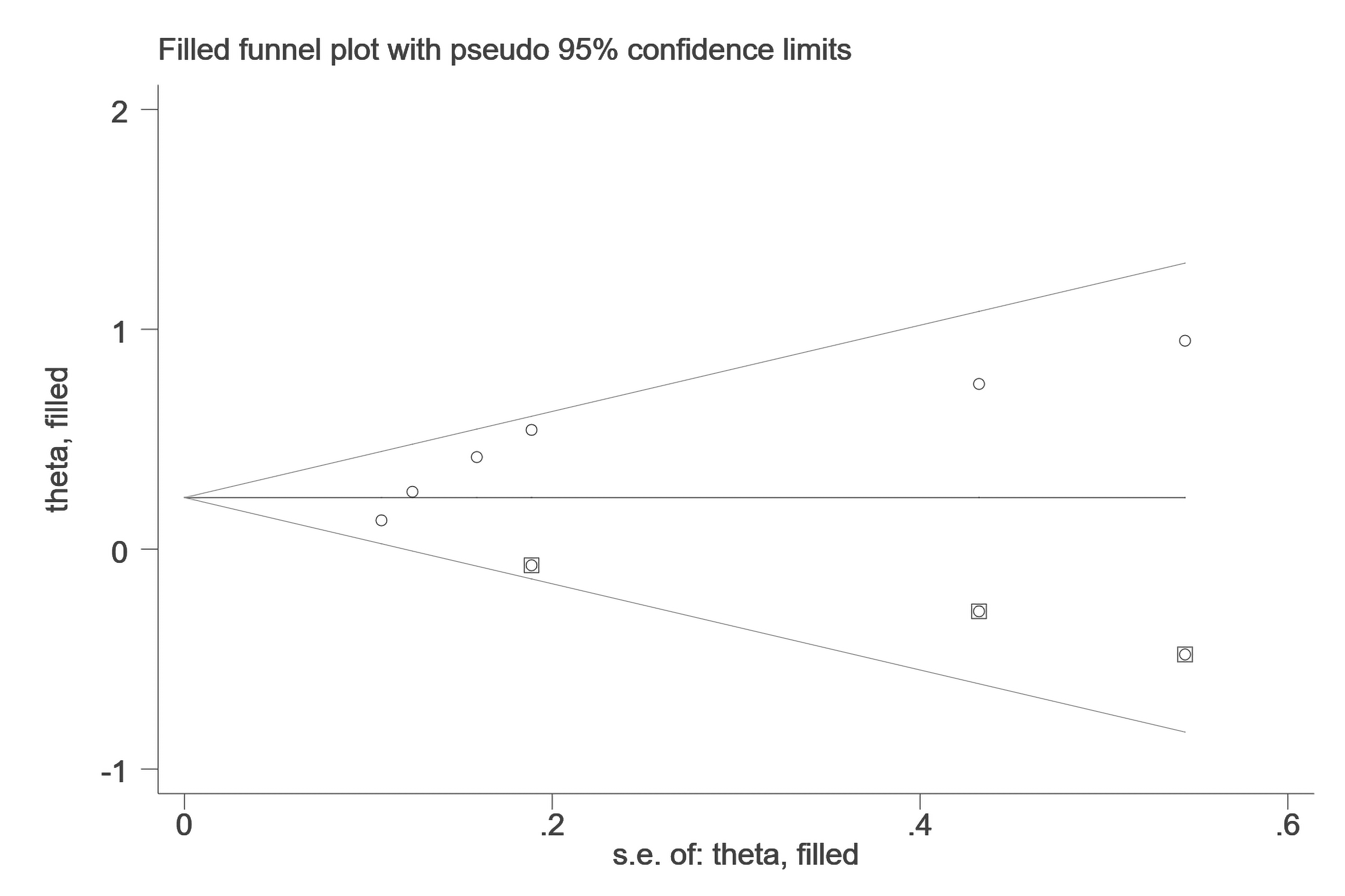

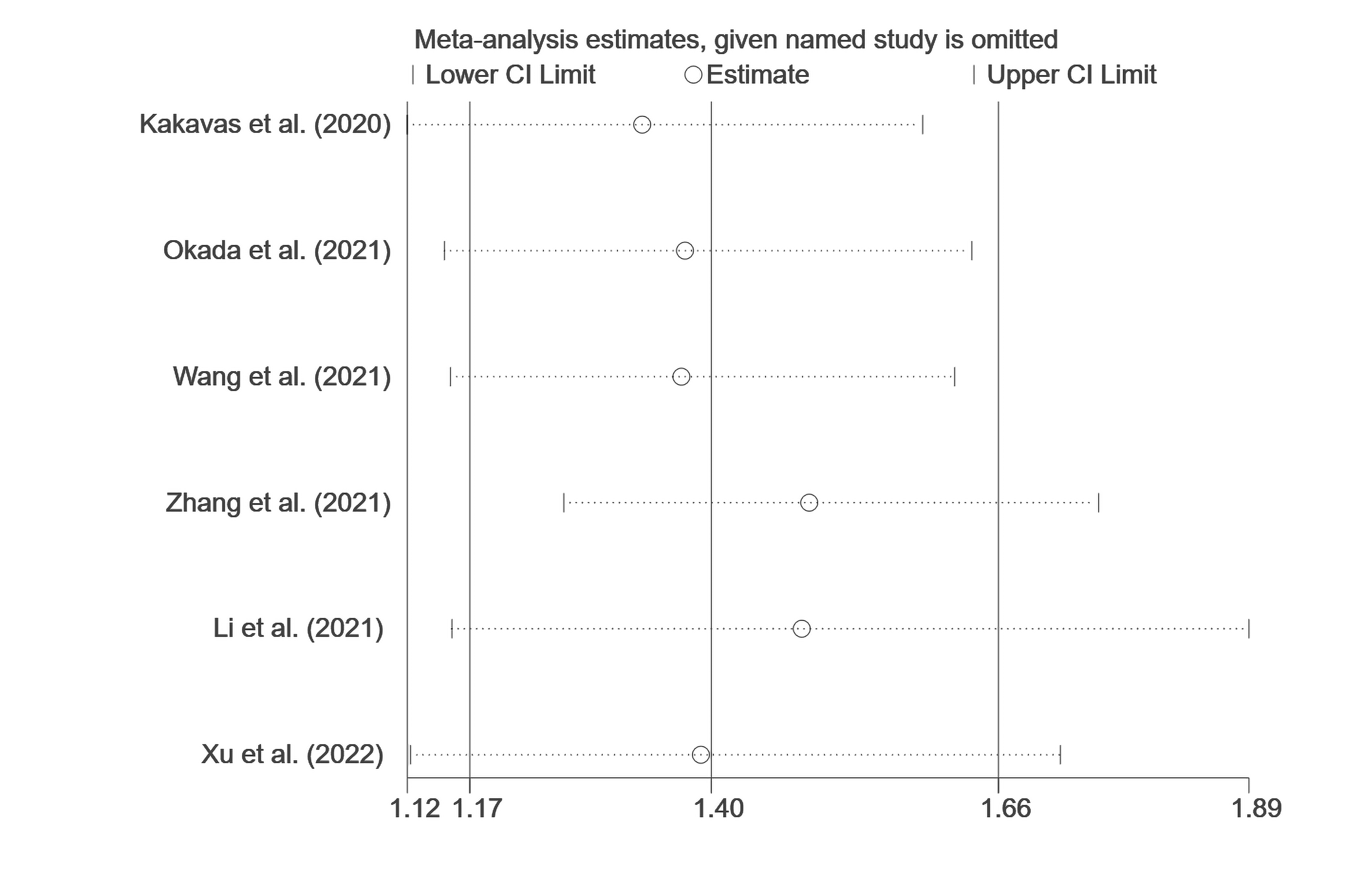


Supplemental Figure 6 Funnel plot showing enrolled studies of overall survival under multivariate regression model (moderately malnourished vs well-nourished). The circles alone are real studies and the circles enclosed in boxes are "imputed" studies.

Supplemental Figure 5 Sensitive analysis for pooled result of overall survival under multivariate regression model (moderately malnourished vs well-nourished). The circles represent the pooled results after removing individual study. “|” represent the lower and upper 95%CI limit.


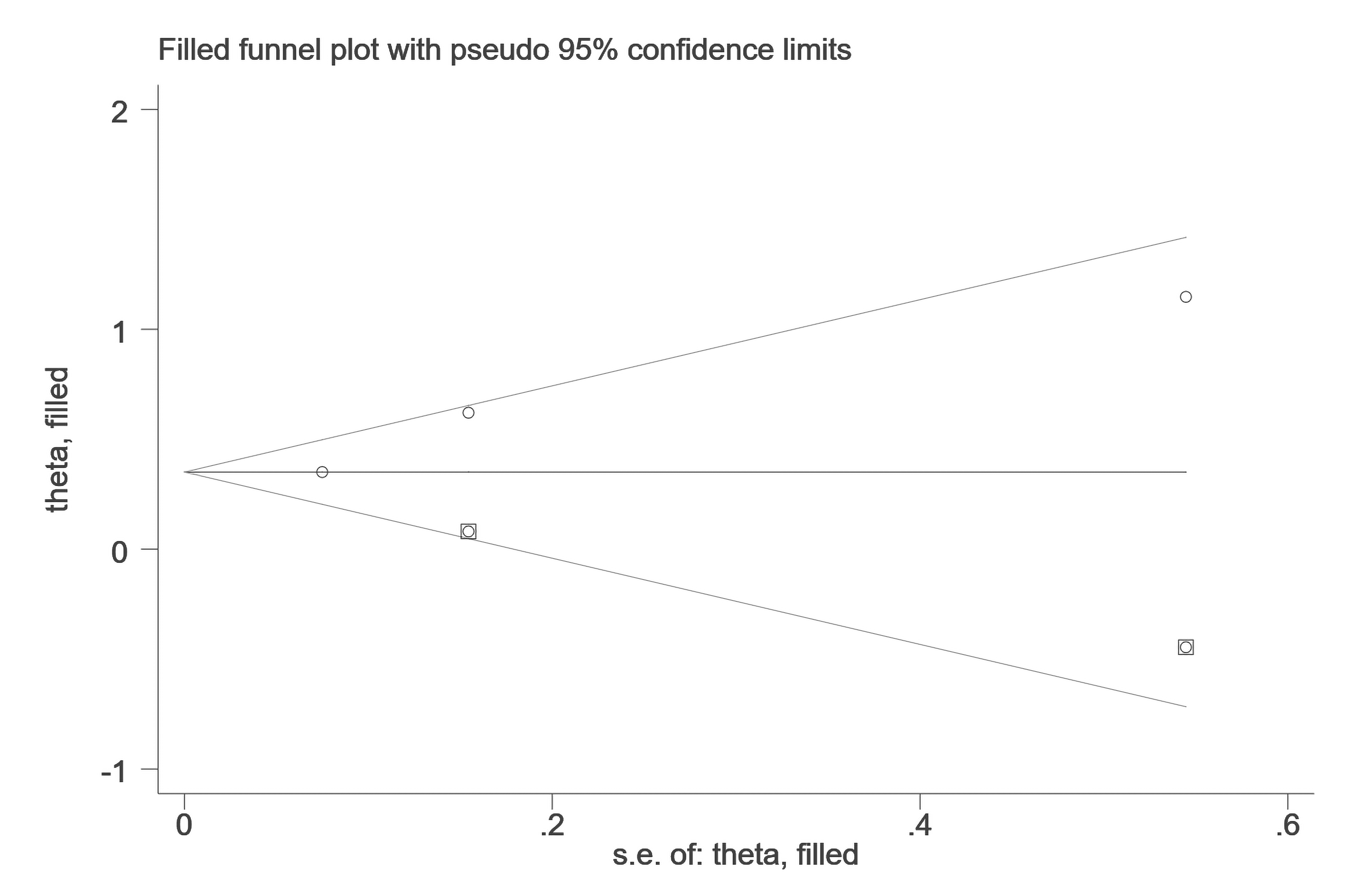

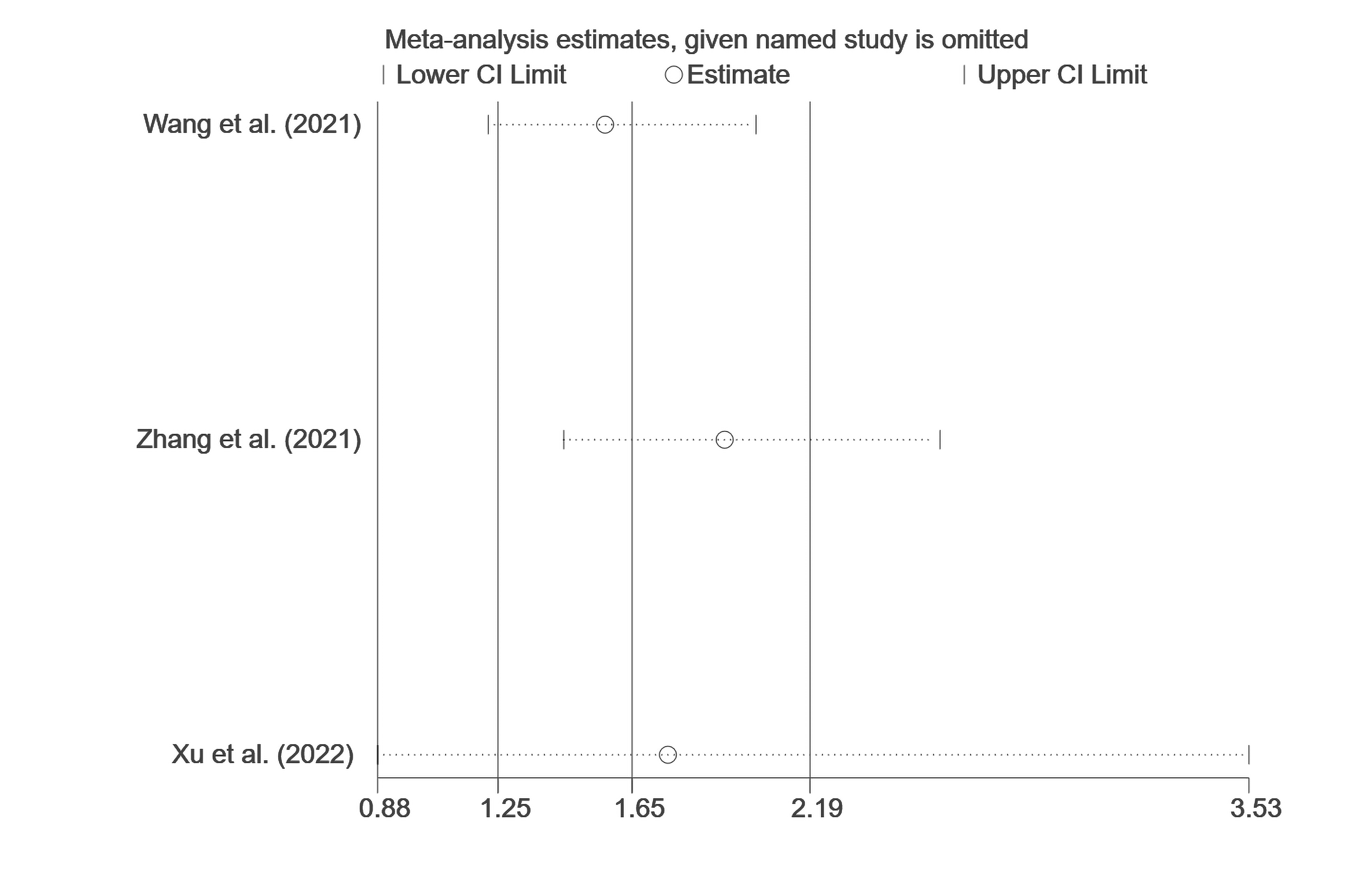


Supplemental Figure 7 Sensitive analysis for pooled result of overall survival under univariate regression model (moderately malnourished vs well-nourished). The circles represent the pooled results after removing individual study. “|” represent the lower and upper 95%CI limit.

Supplemental Figure 8 Funnel plot showing enrolled studies of overall survival under univariate regression model (moderately malnourished vs well-nourished). The circles alone are real studies and the circles enclosed in boxes are "imputed" studies.


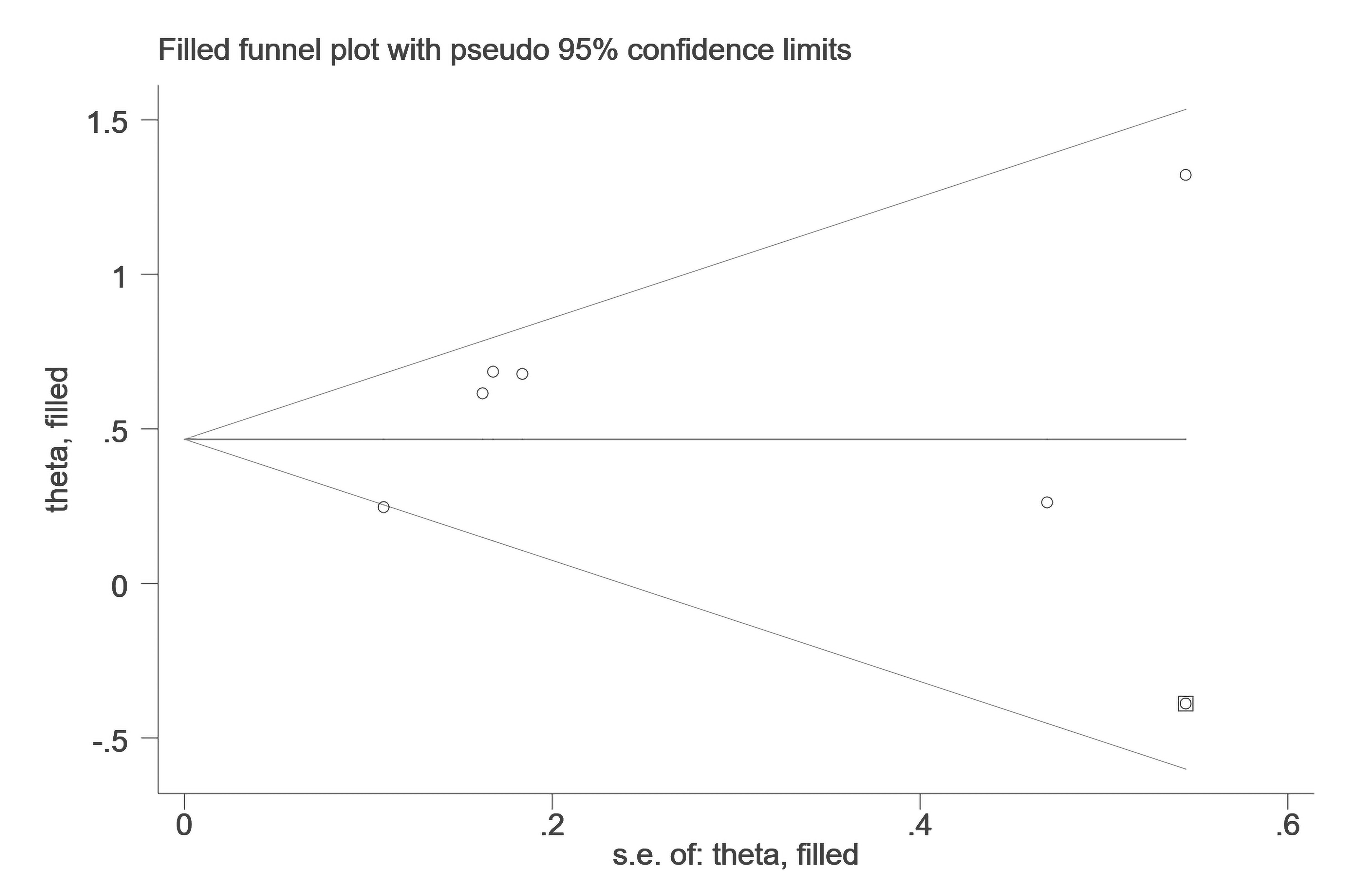

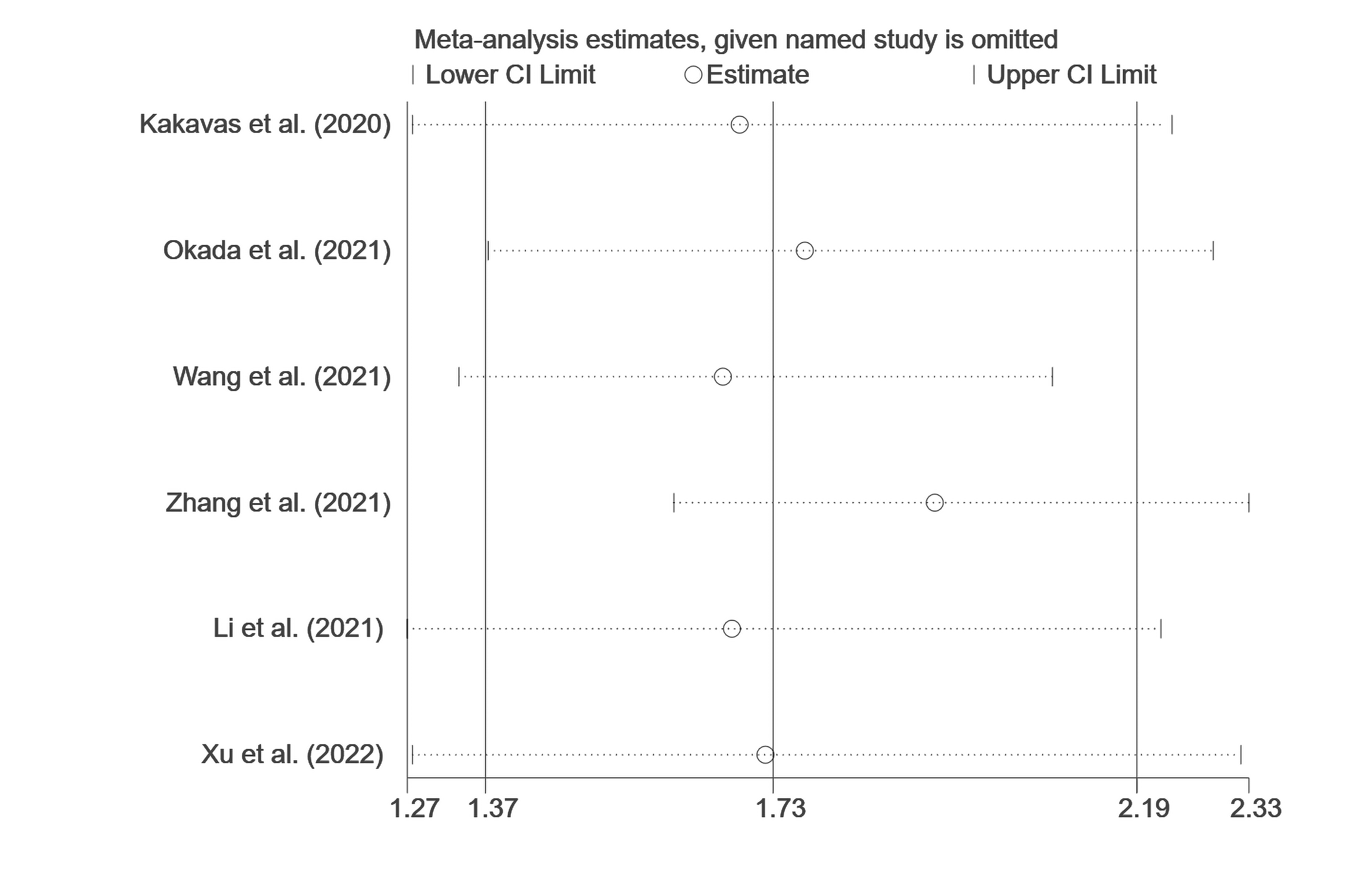


Supplemental Figure 10 Funnel plot showing enrolled studies of overall survival under multivariate regression model (severely malnourished vs well-nourished). The circles alone are real studies and the circles enclosed in boxes are "imputed" studies.

Supplemental Figure 9 Sensitive analysis for pooled result of overall survival under multivariate regression model (severely malnourished vs well-nourished). The circles represent the pooled results after removing individual study. “|” represent the lower and upper 95%CI limit.


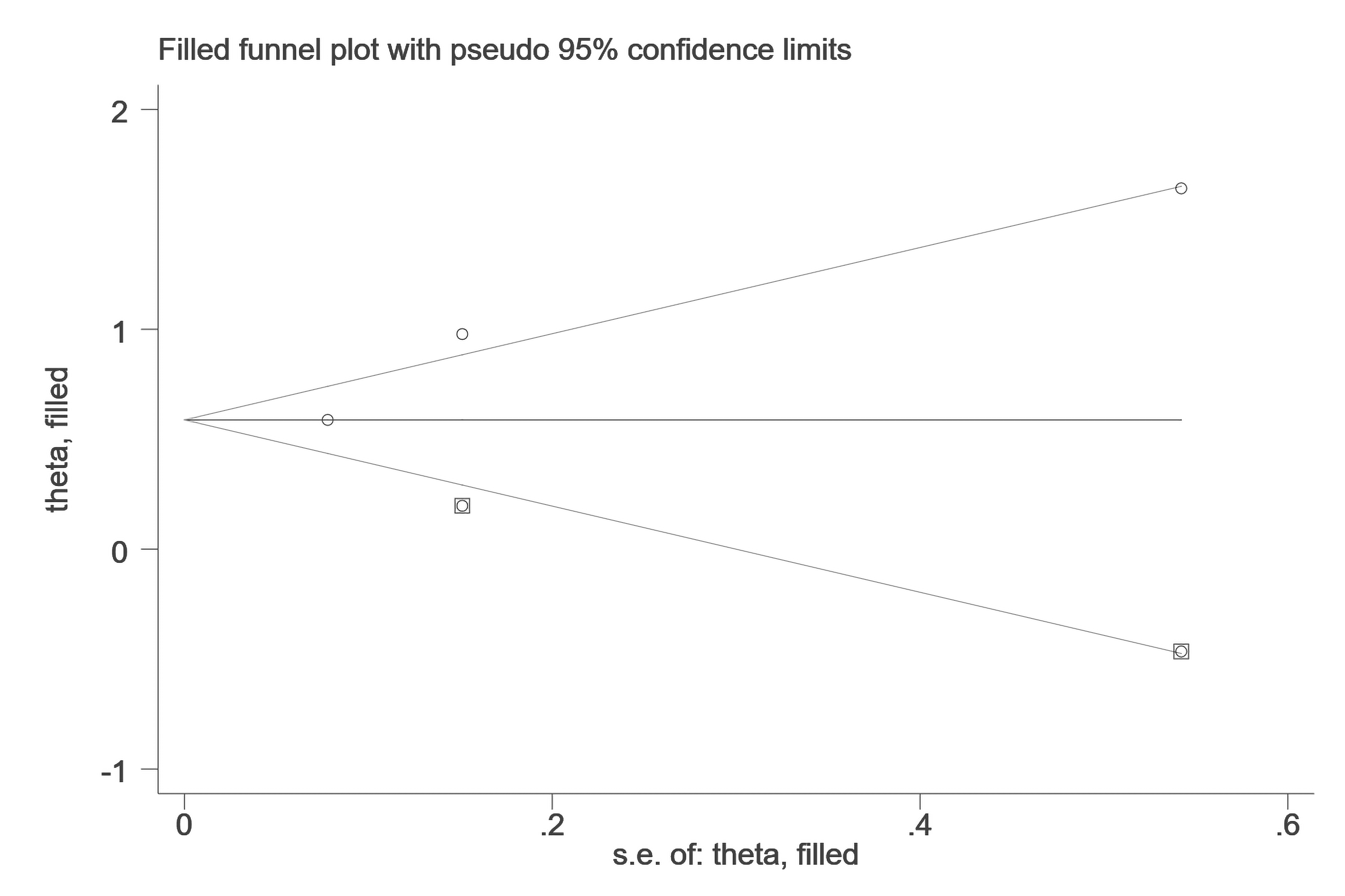

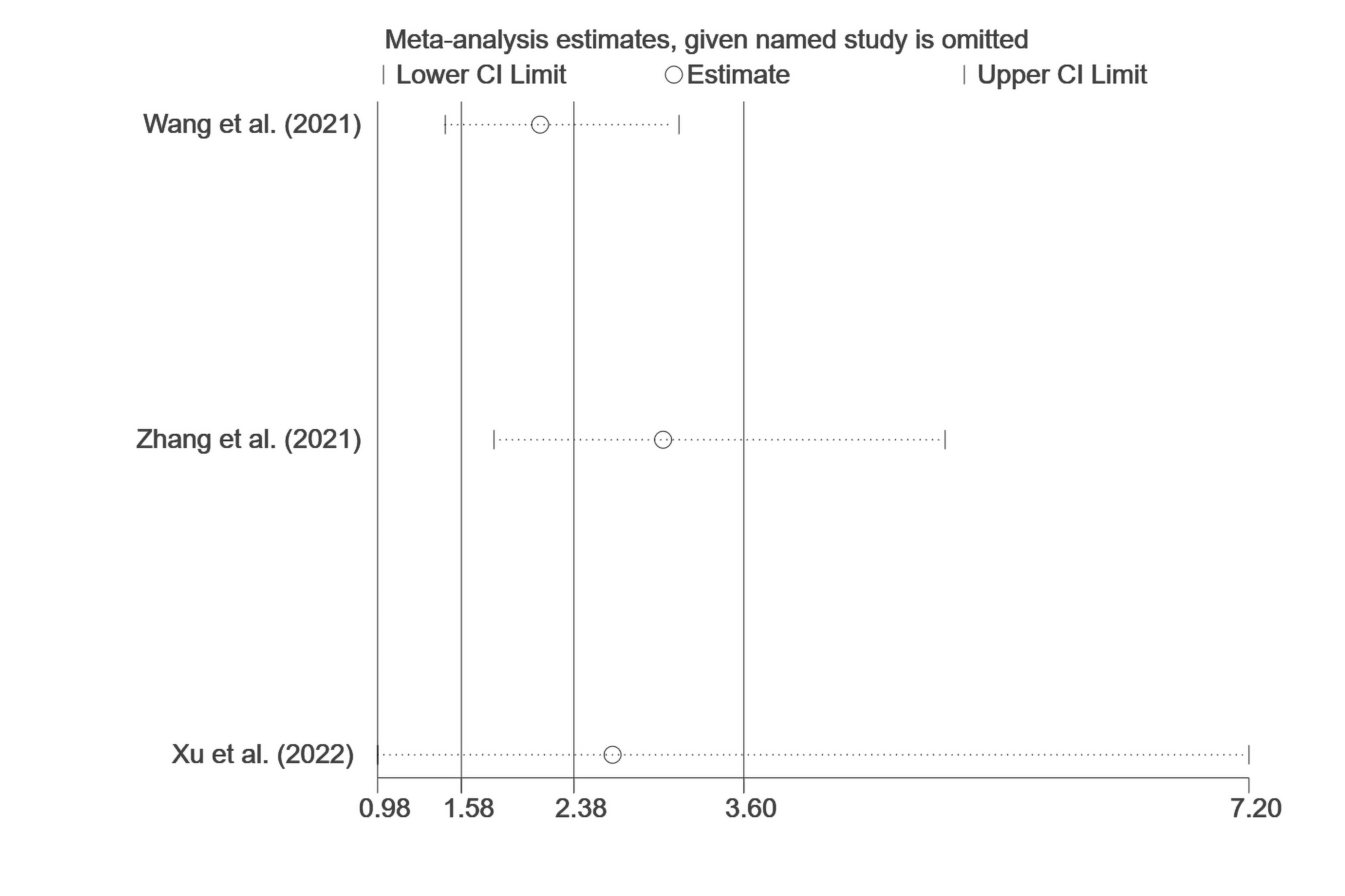


Supplemental Figure 12 Funnel plot showing enrolled studies of overall survival under univariate regression model (severely malnourished vs well-nourished). The circles alone are real studies and the circles enclosed in boxes are "imputed" studies.

Supplemental Figure 11 Sensitive analysis for pooled result of overall survival under univariate regression model (severely malnourished vs well-nourished). The circles represent the pooled results after removing individual study. “|” represent the lower and upper 95%CI limit.


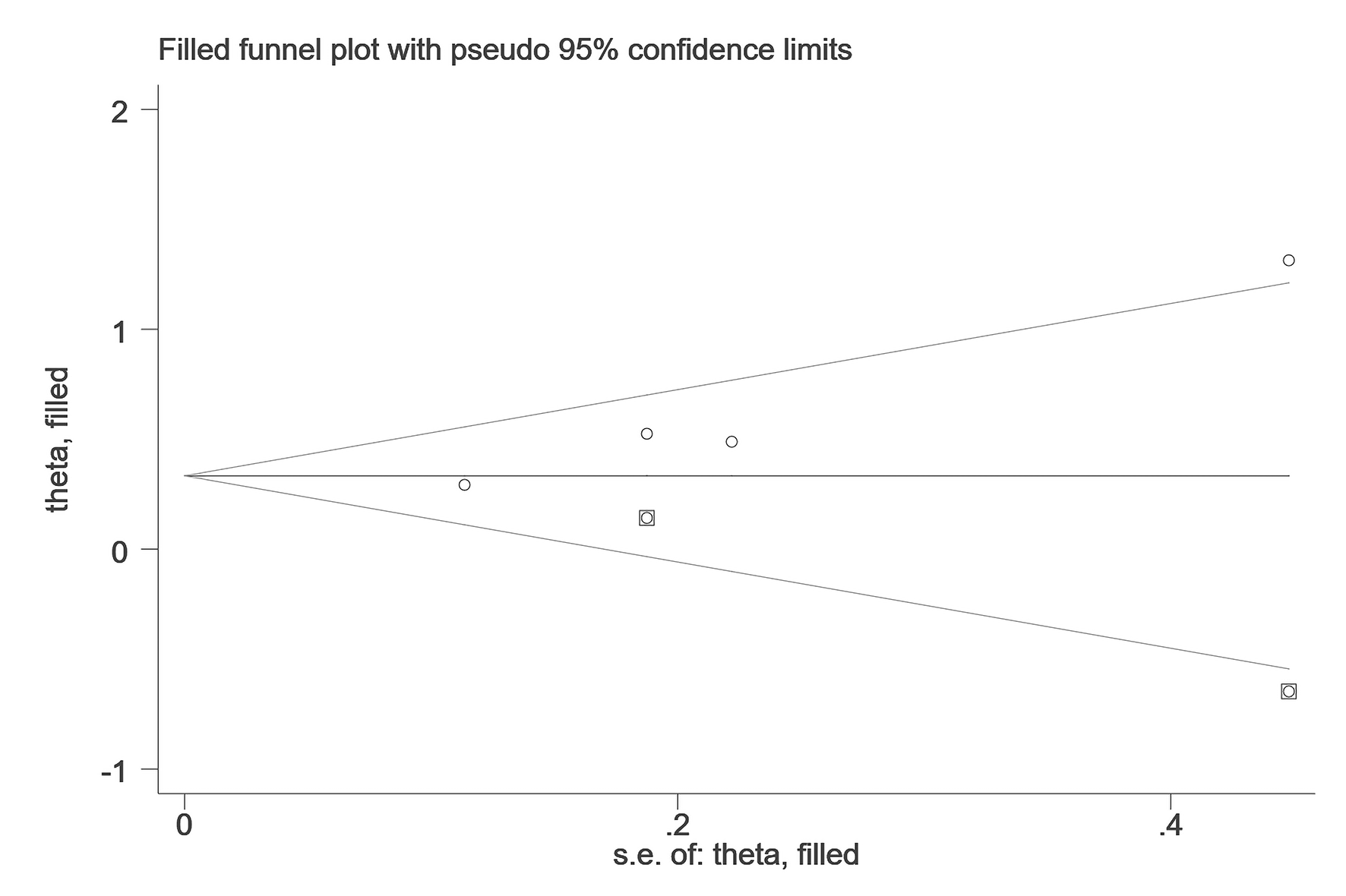

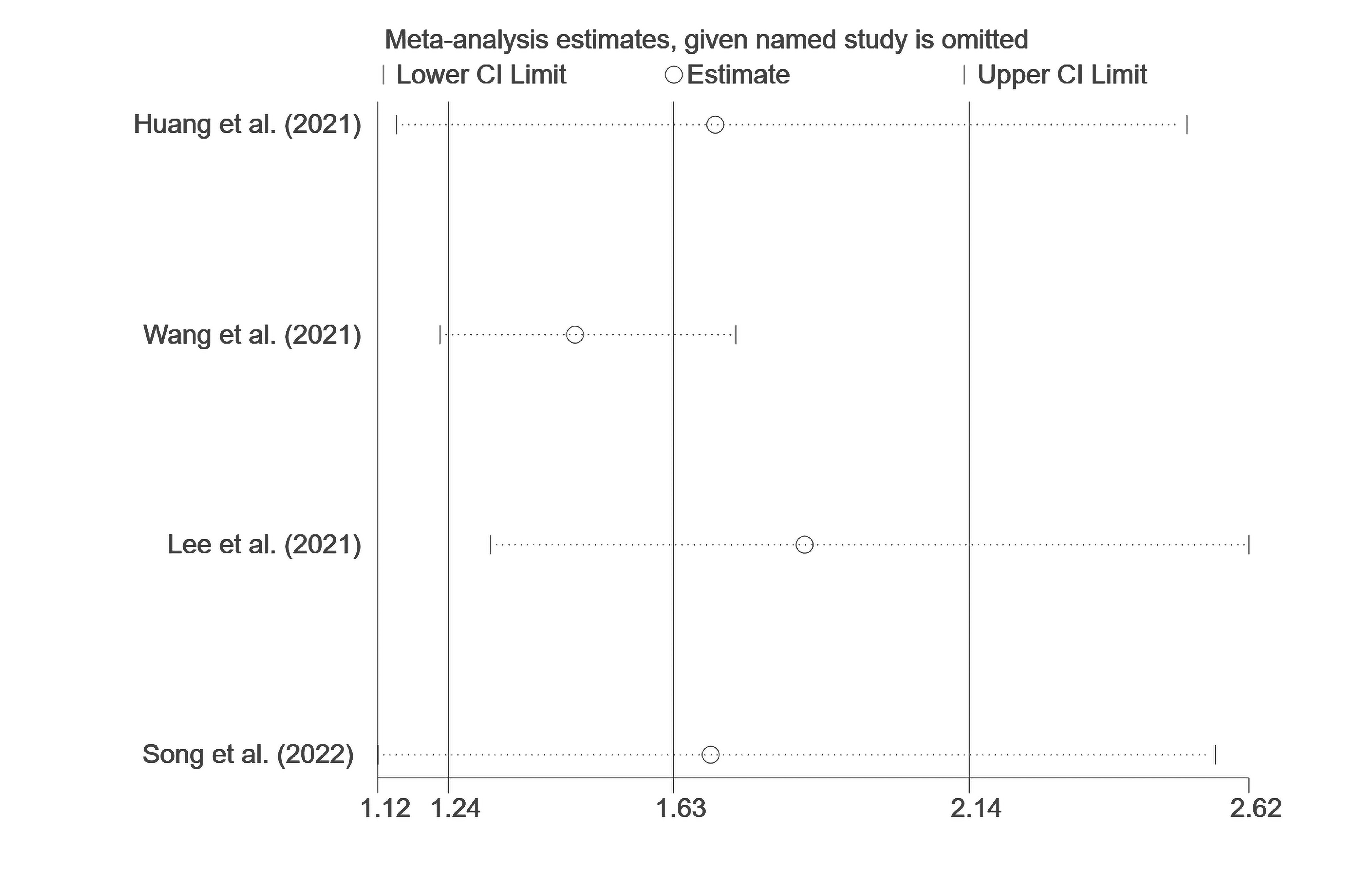


Supplemental Figure 13 Sensitive analysis for pooled result of disease-free survival under multivariate regression model (malnourished vs well-nourished). The circles represent the pooled results after removing individual study. “|” represent the lower and upper 95%CI limit.

Supplemental Figure 14 Funnel plot showing enrolled studies of disease-free survival under univariate regression model (malnourished vs well-nourished). The circles alone are real studies and the circles enclosed in boxes are "imputed" studies.


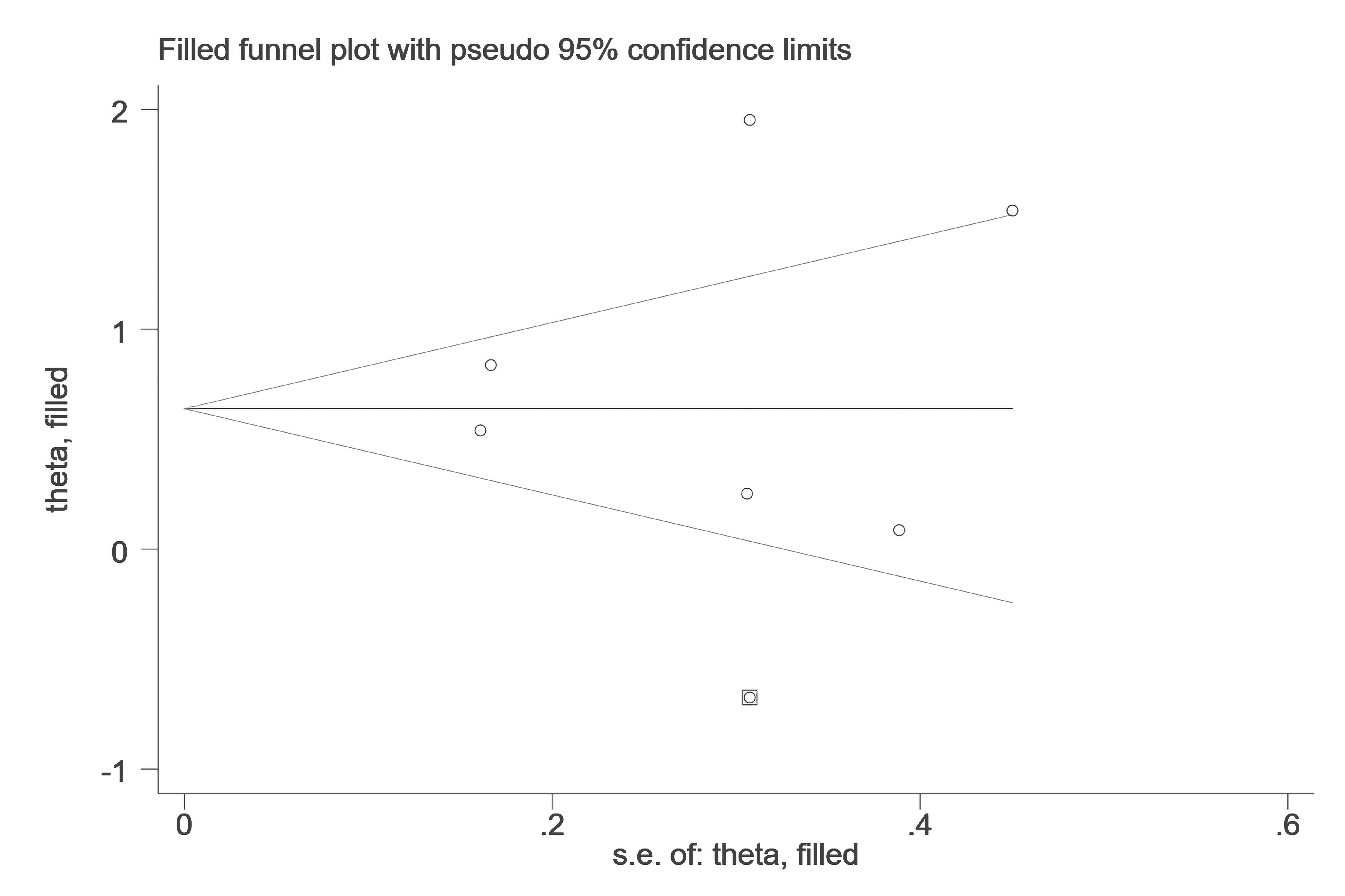

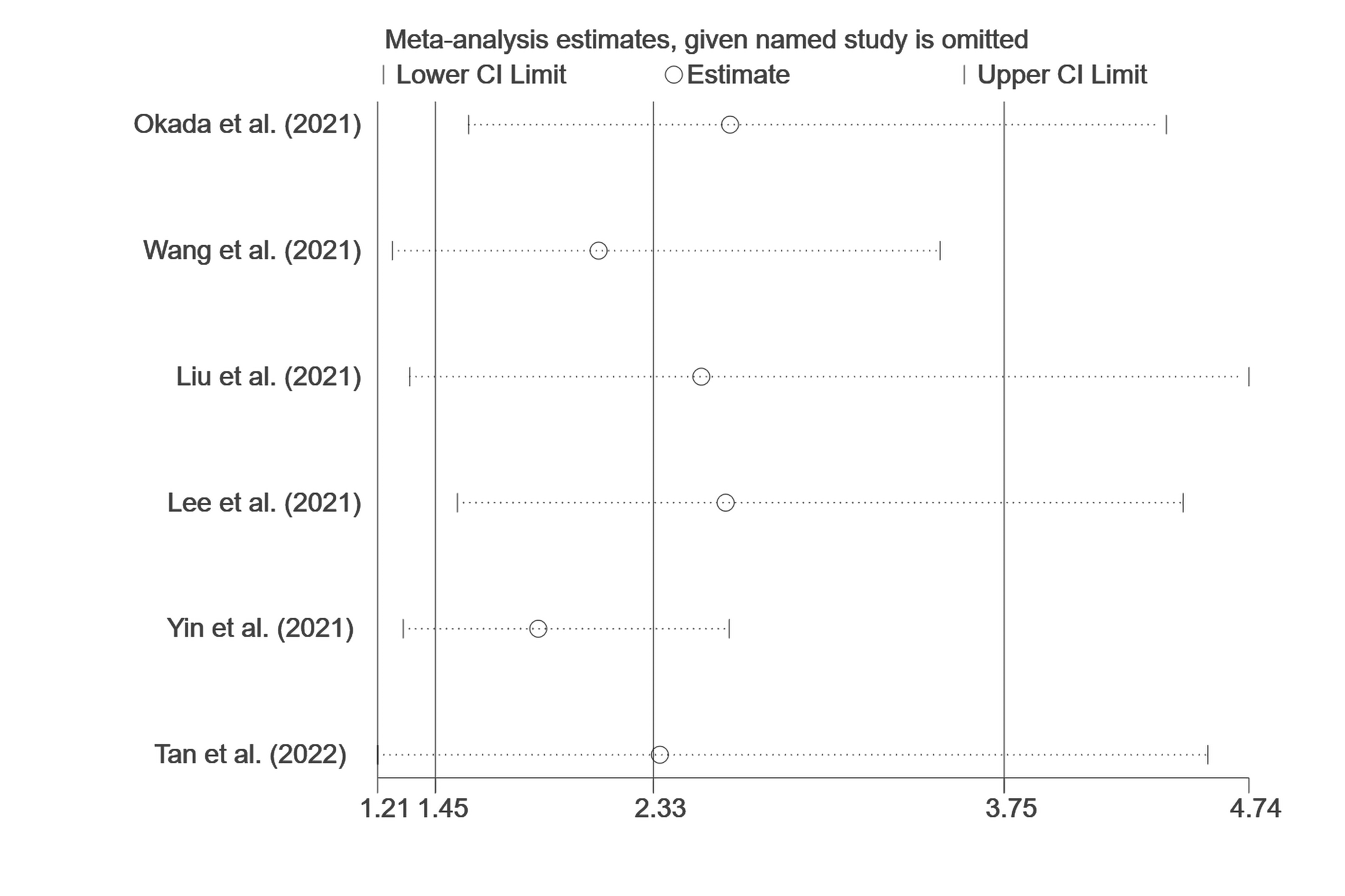


Supplemental Figure 16 Funnel plot showing enrolled studies of overall complications under univariate regression model (malnourished vs well-nourished). The circles alone are real studies and the circles enclosed in boxes are "imputed" studies.

Supplemental Figure 15 Sensitive analysis for pooled result of overall complications under univariate regression model (malnourished vs well-nourished). The circles represent the pooled results after removing individual study. “|” represent the lower and upper 95%CI limit.


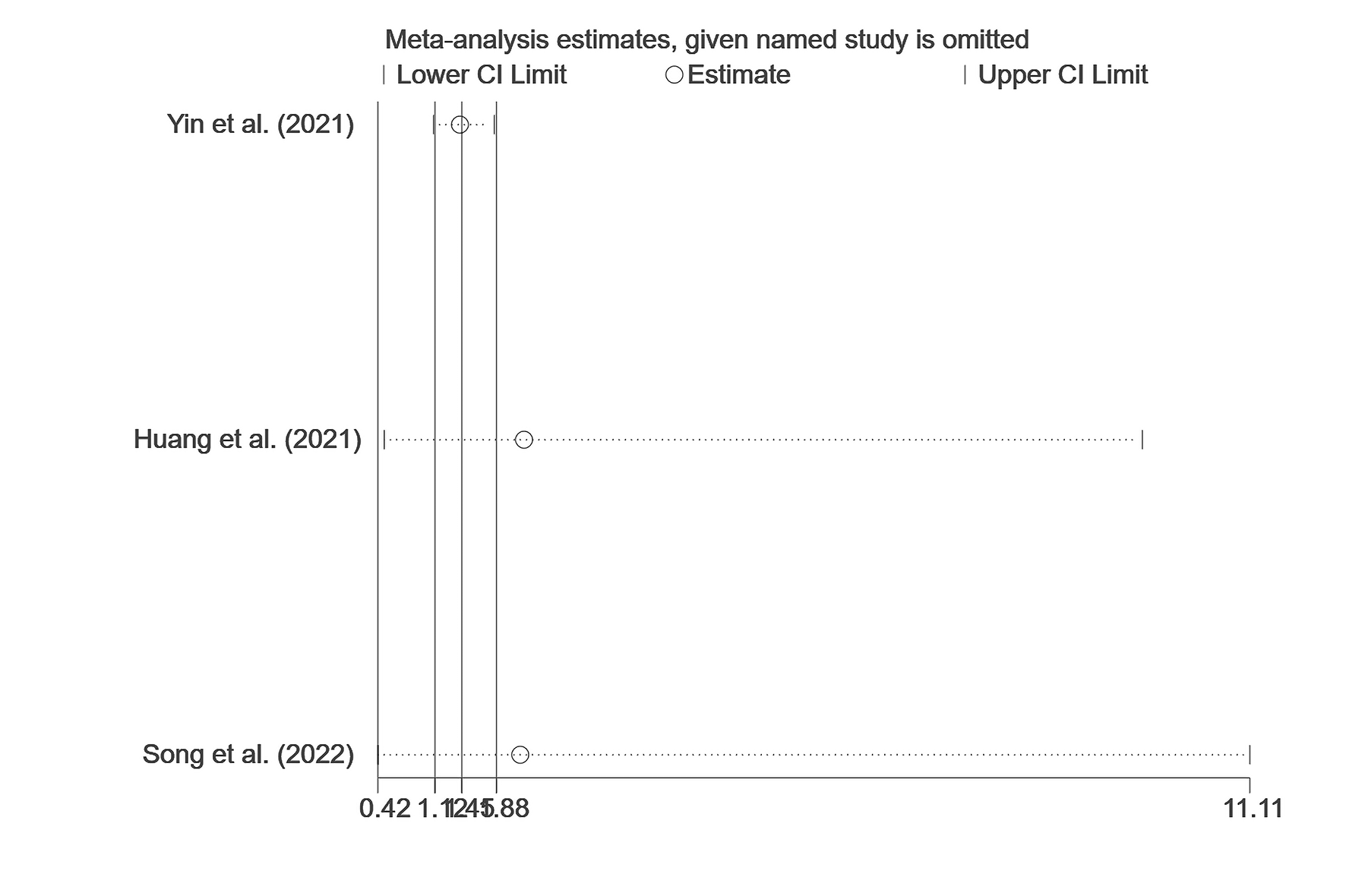


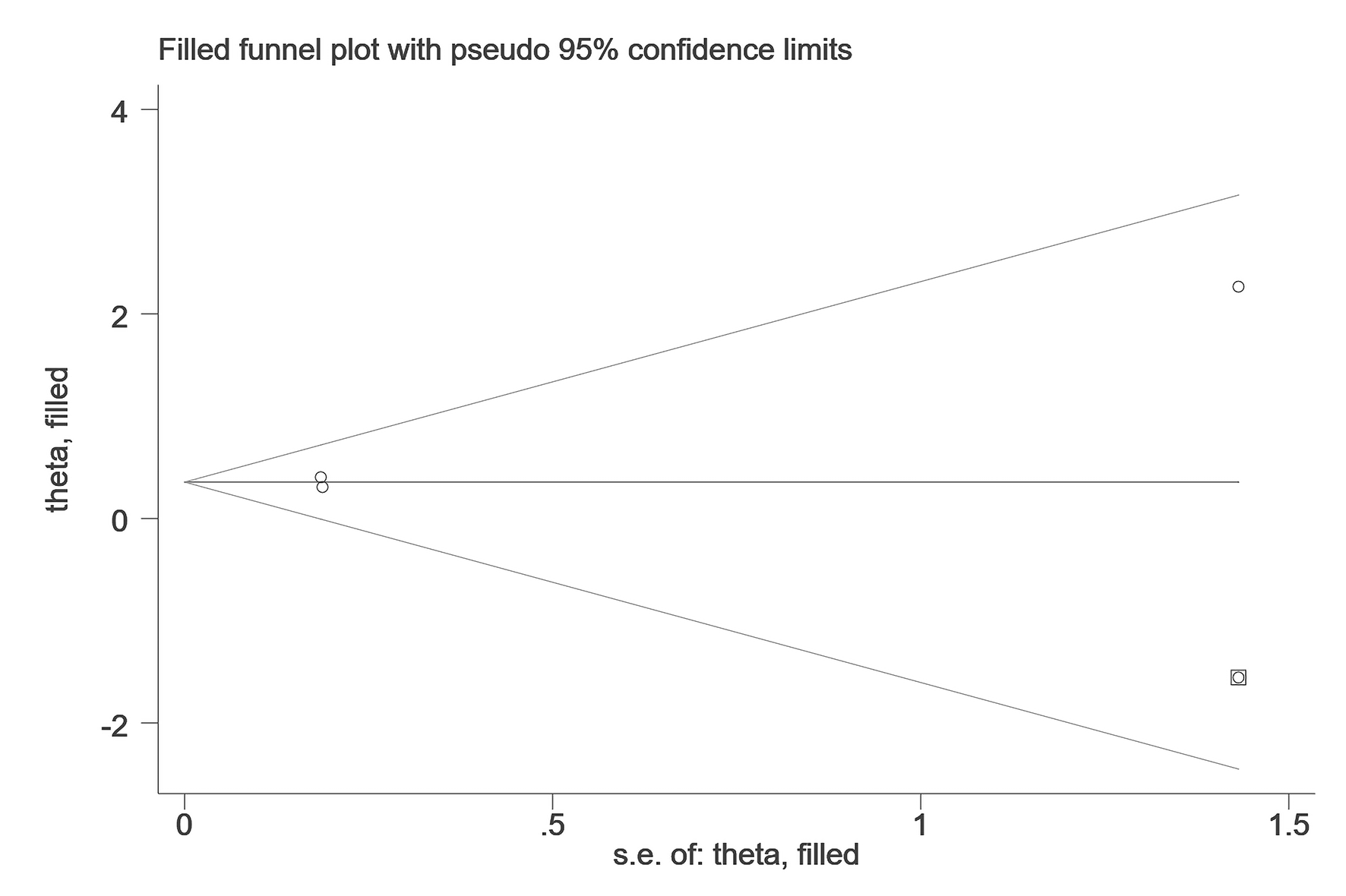


Supplemental Figure 18 Funnel plot showing enrolled studies of complications ≥ Clavien-Dindo grade IIa under univariate regression model (malnourished vs well-nourished). The circles alone are real studies and the circles enclosed in boxes are "imputed" studies.

Supplemental Figure 17 Sensitive analysis for pooled result of complications ≥ Clavien-Dindo grade IIa under univariate regression model (malnourished vs well-nourished). The circles represent the pooled results after removing individual study. “|” represent the lower and upper 95%CI limit.


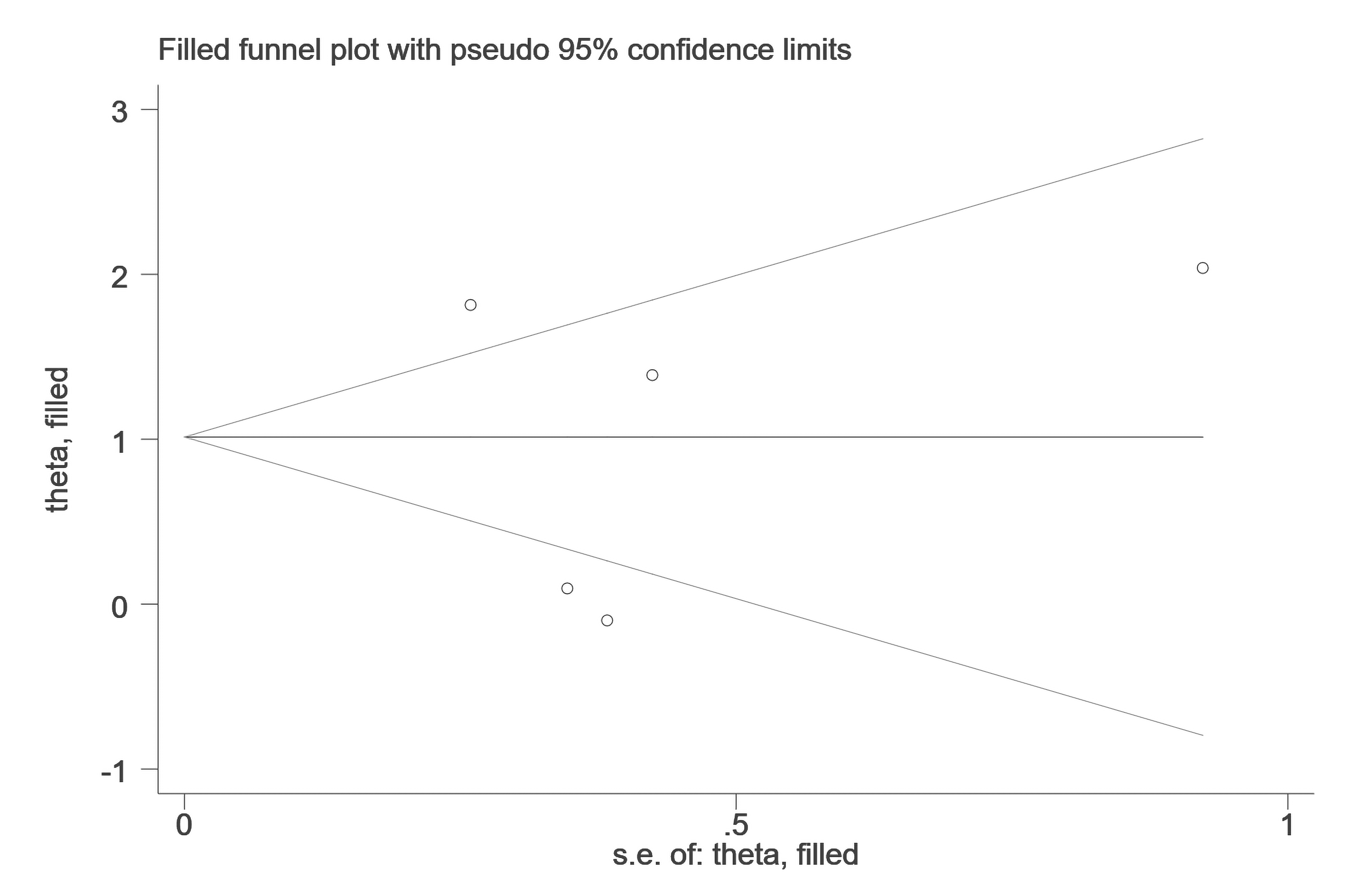

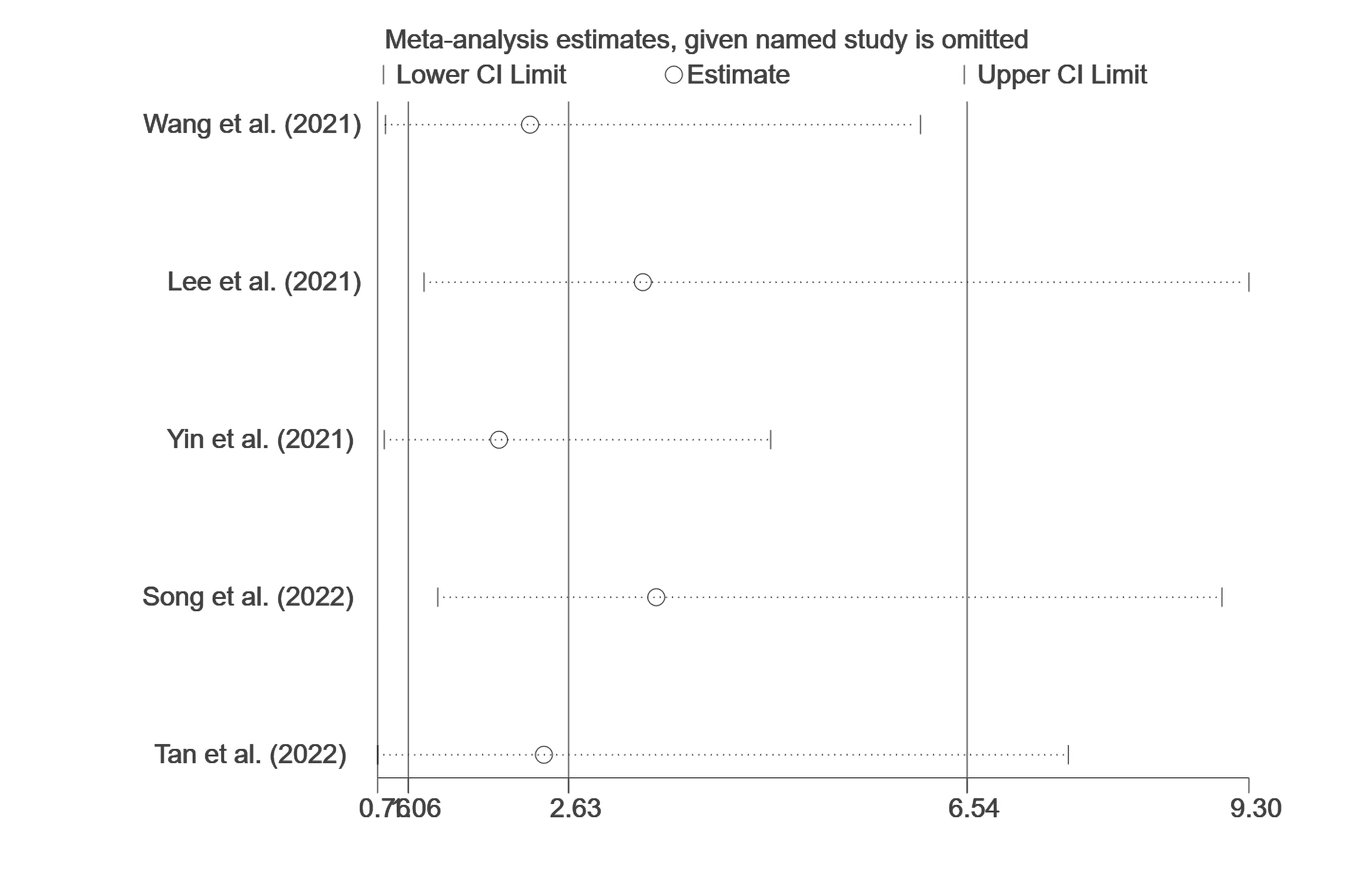


Supplemental Figure 20 Funnel plot showing enrolled studies of complications ≥ Clavien-Dindo grade IIIa under univariate regression model (malnourished vs well-nourished). The circles alone are real studies and the circles enclosed in boxes are "imputed" studies.

Supplemental Figure 19 Sensitive analysis for pooled result of complications ≥ Clavien-Dindo grade IIIa under univariate regression model (malnourished vs well-nourished). The circles represent the pooled results after removing individual study. “|” represent the lower and upper 95%CI limit.
